# Elucidation of intragenomic variation of ribosomal DNA sequences in the enigmatic fungal genus *Ceraceosorus*, including a newly described species *Ceraceosorus americanus*

**DOI:** 10.1186/s43008-024-00172-7

**Published:** 2024-12-30

**Authors:** Teeratas Kijpornyongpan, Mary Claire Noble, Marcin Piątek, Matthias Lutz, M. Catherine Aime

**Affiliations:** 1 Department of Botany and Plant Pathology, Purdue University, West Lafayette, IN USA; 2 W. Szafer Institute of Botany, Polish Academy of Sciences, Kraków, Poland; 3 Plant Evolutionary Ecology, Institute of Evolution and Ecology, University of Tübingen, Tübingen, Germany

**Keywords:** Concerted evolution, Exobasidiomycetes, Internal transcribed spacer, 1 new taxon, Nucleotide polymorphism, Sanger sequencing, Smut fungi, WGS sequencing

## Abstract

Multicopy nuclear ribosomal DNA (rDNA) genes have been used as markers for fungal identification for three decades. The rDNA sequences in a genome are thought to be homogeneous due to concerted evolution. However, intragenomic variation of rDNA sequences has recently been observed in many fungi, which may make fungal identification and species abundance estimation based on these loci problematic. *Ceraceosorus* is an enigmatic genus in the smut lineage *Ustilaginomycotina* for which very limited distribution data exist. Our previous research demonstrated intragenomic variation in the internal transcribed spacer (ITS1-5.8S-ITS2) region of two *Ceraceosorus* species. In this study, we described the fourth known species of *Ceraceosorus*, *C. americanus*, isolated from an asymptomatic rosemary leaf collected in Louisiana, USA. This is the first report of this genus in the Americas. We then selected all four known *Ceraceosorus* species, plus exemplar smut fungi representing all major lineages of subphylum *Ustilaginomycotina*, to examine sequence heterogeneity in three regions of the rDNA repeat (partial 18S, ITS, and partial 28S regions). Three methods were used: PCR-cloning-Sanger sequencing, targeted amplicon high-throughput sequencing, and whole-genome shotgun high-throughput sequencing. Our results show that *Ceraceosorus* is the only sampled fungal genus in *Ustilaginomycotina* with significant intragenomic variation at the ITS, with up to 25 nucleotide variant sites in the ITS1–5.8S–ITS2 region and 2.6% divergence among analyzed ITS haplotypes. We found many conflicting patterns across the three detection methods, with up to 27 conflicting variant sites recorded from a single individual. At least 40% of the conflicting patterns are possibly due to PCR-cloning-sequencing errors, as the corresponding variant sites were not observed in the other detection methods. Based on our data and the literature, we evaluated the characteristics and advantages/disadvantages of each detection method. Finally, a model for how intragenomic variation in the rDNA copies within a genome may arise is presented.

**Supplementary Information:**

The online version contains supplementary material available at 10.1186/s43008-024-00172-7.

## INTRODUCTION

*Ceraceosorus* is an enigmatic fungal genus in Ceraceosorales, *Ustilaginomycotina*. It was first described as a non-teliosporic phytopathogen, typified by *Ceraceosorus bombacis* from the leaves of *Bombax ceiba* in India (Bakshi et al. 1972; Cunningham et al. 1976), now placed in the Exobasidiomycetes as a sister to species of the *Entylomatales* (Begerow et al. 2006; Kijpornyongpan et al. 2018). The *Entylomatales* contains mainly teliosporic smuts that infect dicotyledonous hosts and show a high degree of host specificity (Kruse et al. 2018; Piątek et al. 2024; Vánky 2012). The genus *Ceraceosorus* currently consists of three described species: *C. africanus, C. bombacis*, and *C. guamensis* (Cunningham et al. 1976; Kijpornyongpan and Aime 2016; Piątek et al. 2016). *Ceraceosorus bombacis* and *C. africanus* were originally described from the sexual stage as phytopathogens on the leaves of *Bombax* spp., while *C. guamensis* is only known from a yeast-like asexual stage. The yeast-like stage was also isolated and described in *C. bombacis* (Cunningham et al. 1976), while the culturing of *C. africanus* was not attempted. *Ceraceosorus* species are known only from India (*C. bombacis*), Western Africa (*C. africanus*), and the island of Guam in Oceania (*C. guamensis*) (Cunningham et al. 1976; Kijpornyongpan and Aime 2016; Piątek et al. 2016).

Nuclear ribosomal DNA (rDNA) genes are among the most conserved and redundant genes in living organisms. They encode ribosomal RNA, which plays a critical role in ribosome assembly and protein synthesis (Bruns et al. 1991; Hillis and Dixon 1991). In typical eukaryotic genomes, there are four types of encoded rRNAs: 18S, which is part of the small subunit of the ribosome, and 5S, 5.8S, and 28S, which together are part of the large subunit of the ribosome. While the 5S rDNA shows more variability in its presence/absence and its location in the genomes (Drouin and Moniz de Sá, 1995), the rDNA sequences encoding 5.8S, 18S, and 28S rRNA are generally clustered as a single operon unit that is tandemly repeated in the genomes. The copy number of rDNA sequences varies among different animal, plant, and fungal species, ranging from a dozen to tens of thousands (Lofgren et al. 2019; Prokopowich et al. 2003). Although there is a positive correlation between genome size and rDNA copy number in animals and plants (Prokopowich et al. 2003), this trend is not observed in fungi (Lofgren et al. 2019).

In contrast to the mutation-selection mechanisms that generally occur within a genome, most rDNA copies in a genome appear to be homogeneous (Eickbush and Eickbush 2007; Hillis and Dixon 1991; Liao 1999; Nei and Rooney 2005). A phenomenon called “concerted evolution” has been used to explain how most rDNA copies in a genome maintain sequence homogeneity (Eickbush and Eickbush 2007; Elder and Turner 1995; Liao 1999). DNA recombination and gene conversion are two main forces for gene homogenization. This mechanism is clearly reviewed and discussed elsewhere (Eickbush and Eickbush 2007; Liao 1999; Paloi et al. 2022). Recombination-mediated gene homogenization has recently been demonstrated in fungal rDNA sequences (Dakal et al. 2016; Liu et al. 2015). However, many recent studies reveal that intragenomic variation of the rDNA sequences is more common throughout the fungal kingdom than previously thought. Previously, we demonstrated extensive intragenomic variation in the internal transcribed spacer 1–5.8S–internal transcribed spacer 2 (ITS) region of rDNA repeats in two species of *Ceraceosorus*: *C. bombacis* and *C. guamensis* (Kijpornyongpan and Aime 2016). However, the intragenomic variation in other *Ustilaginomycotina* species, as well as in other rDNA regions have yet to be investigated.

In the present study we describe the new species *C. americanus*, which represents the first record of the genus in the Americas. Then we investigate intragenomic rDNA variation in all four described species of *Ceraceosorus* and from representatives of additional lineages in *Ustilaginomycotina*. We employed three methods—PCR-cloning-Sanger sequencing, targeted amplicon high-throughput sequencing, and whole-genome shotgun (WGS) high-throughput sequencing—to sequence the rDNA repeat. We then compare detected variants across the three methods to determine consensus variant sites, to cross-check for data consistency and accuracy, and to determine technical challenges for each detection method. Finally, a model for the origin of rDNA sequence heterogeneity is proposed and discussed.

## MATERIALS AND METHODS

### Sample used for this study

The fungal strain SA252w was isolated from an abaxial surface of an asymptomatic leaf of *Rosmarinus officinalis* (*Lamiaceae*) collected on the campus of Louisiana State University, Baton Rouge, Louisiana, USA. Isolation followed the spore drop method described in Albu et al. (2015). The culture was stored on potato dextrose agar (PDA) slants at 4 °C and in 15% glycerol at − 80 °C. A dried culture plate was vouchered in the Kriebel Herbarium, Purdue University (PUL) under the voucher number F22392. An ex-type culture has been deposited in the Agricultural Research Service (USDA-ARS) Culture Collection and the Westerdijk Fungal Biodiversity Institute Culture Collection (CBS) under accession nos. NRRL 66778 and CBS 144261, respectively.

Other *Ceraceosorus* strains/specimens included in this study were as follows: the culture collection nos. ATCC 22867 and MCA4658 (≡ CBS 139631 ≡ NRRL 66309) for the ex-type strains of *C. bombacis* (from the leaves of *Bombax ceiba* in India) and *C. guamensis* (from the surface of a healthy dicot leaf in Guam), respectively, and KRAM F-57385, KRAM F-57386, and KRAM F-57387 for the vouchered specimens of *C. africanus* (from the leaves of *Bombax costatum* in Benin and Ghana), deposited at the fungal collection of the W. Szafer Institute of Botany, Polish Academy of Sciences, Kraków.

We also included several other species representing major lineages in *Ustilaginomycotina* *fide* Kijpornyongpan et al. (2018): *Testicularia cyperi* MCA3645 (≡ ATCC MYA-4640) and *Mycosarcoma maydis* (syn. *Ustilago maydis*) TKC58 (≡ NRRL Y-64004) for *Ustilaginales*, *Meira miltonrushii* MCA3882 (≡ CBS 12591) and *Laurobasidium hachijoense* (syn. *Acaromyces ingoldii*) MCA4198 (≡ CBS 140884) for *Exobasidiales*, *Tilletiopsis washingtonensis* MCA4186 (≡ NRRL Y-63783) for *Entylomatales*, *Pseudomicrostroma glucosiphilum* MCA4718 (≡ CBS 14053) and *Jaminaea rosea* MCA5214 (≡ CBS 14051) for *Microstromatales*, *Violaceomyces palustris* SA807 (≡ CBS 139708) for *Violaceomycetales*, and *Tilletiaria anomala* CBS 436.72 for *Georgefischeriales*.

### Culture description

To observe colony morphology, the strain SA252w was cultured on four types of media: PDA, corn meal agar (CMA), yeast malt agar (YMA), and yeast peptone glucose agar (YPGA). The cultures were incubated at room temperature (25 °C) for 14 days. Colony color was compared to color codes from The Online Auction Color Chart™ (Online Auction Color Chart 2004). For cell morphology, the strain was inoculated in yeast malt broth (YMB) and incubated for 7 days at room temperature and 200 round-per-minute shaking condition. A drop of the culture suspension was then placed on a microscope slide, followed by a coverslip. We observed cell morphology using an Olympus BH2 microscope in differential interference contrast mode. Photomicrographs were captured by QImaging with QI Imaging software. Cell sizes were measured for a minimum of 30 cells. The physiological profiles of SA252w were examined using a protocol described in a previous study (Kijpornyongpan and Aime 2016).

### DNA extraction, rDNA amplification and sequencing

DNA was extracted from fresh cultures growing on PDA at room temperature. A piece of colony was cut for extraction using the Omega fungal DNA HP kit (Norcross, GA, USA) following the manufacturer’s protocol. For the vouchered specimens of *C. africanus,* DNA was extracted as described in Piątek et al. (2016).

A variety of primers were used to amplify consensus sequences in different rDNA regions: LR0R/LR6 primers (Vilgalys et al. 1994; Vilgalys and Hester 1990) for partial 28S large subunit rDNA, and PNS1/NS6 primers (Hibbett 1996; White et al. 1990) for partial 18S small subunit rDNA. For the ITS region, we used ITS1Cb (5’- CTT GCT GGC CCG GAG GAA GTA A-3’, designed in this study) and ITS1F (Gardes and Bruns 1993) as forward primers for *Ceraceosorus* and other fungal genera, respectively, and ITS4 (White et al. 1990) as a reverse primer for all studied species. We designed the ITS1Cb primer to specifically match the ITS region of *Ceraceosorus*, which cannot be captured by most commonly employed fungal forward ITS primers (Kijpornyongpan and Aime 2016). The Promega 2X master mix solution with *Taq* DNA polymerase (Promega, Madison, WI) was used as a mixture solution for PCR. Amplification conditions were set as follows: initial denaturation at 95 °C for 5 min, 35 amplification cycles of denaturation at 95 °C for 1 min, annealing at 50 °C for 45 s (ITS region), 50 °C for 1 min (partial 28S region), or 58 °C for 1 min (partial 18S region), and extension at 72 °C for 1 min,﻿ and final extension at 72 °C for 7 min. PCR products were run on 1% agarose gel electrophoresis to determine size and quality. The total PCR products were sent to GENEWIZ (Plainfield, NJ, USA) for direct Sanger sequencing. Raw ab1 files were used to retrieve consensus rDNA sequences using Sequencher 5.2.3 (Gene Code, Ann Arbor, MI, USA).

Due to unreadable chromatograms of ITS amplicons in all samples of *C. africanus* and *Ceraceosorus* sp. SA252w, we suspected ITS intragenomic variation in both species. We therefore performed cloning-Sanger sequencing as described in Kijpornyongpan and Aime (2016). At least 10 clones were sequenced for each strain/specimen.

### Phylogenetic analyses

We first performed a species tree phylogeny to determine species identity of the strain SA252w. The rDNA sequences from all described *Ceraceosorus* species and representative fungi of *Ustilaginomycotina* (see above) were retrieved from the literature or otherwise sequenced in this study (Table 1). *Mixia osmundae* was selected as an outgroup based on its placement in *Pucciniomycotina*, a sister subphylum to *Ustilaginomycotina* (Prasanna et al. 2020). Sequences of each rDNA region were aligned using the MUSCLE algorithm performed in MEGA-X (Kumar et al. 2018). Flanking alignment regions at 5’ and 3’ were manually trimmed. The trimmed alignments of all three rDNA regions were then concatenated and used for phylogenetic reconstruction, which was performed through RAxML 8.2.9 (Stamatakis 2014) with GTRCAT as a nucleotide substitution model and 1000-replicate bootstrapping.

**Table 1:** rDNA sequences used in this study

Species	Sequence accessions			References
	18S	ITS	28S	
*Ceraceosorus americanus* SA252w^T^	KM591596	OQ540449* –OQ540477	KM591595	Albu et al. (2015); This study
*C. africanus* KRAM F-57385^T^	KP413055	OQ540416* –OQ540427	KP413036	Piątek et al. (2016); This study
*C. africanus* KRAM F-57386	–	OQ540403–OQ540415	–	This study
*C. africanus* KRAM F-57387	–	OQ540428* –OQ540448	KP413035	Piątek et al. (2016); This study
*C. bombacis* ATCC 22867^T^	KP413054	KT984941* –KT984950	KP413033	Kijpornyongpan and Aime (2016); Piątek et al. (2016)
*C. guamensis* MCA4658^T^	KT984925	KT984926* –KT984940	KT984924	Kijpornyongpan and Aime (2016)
*Jaminaea rosea* MCA5214^T^	OQ516982	KR912071	KR912073	Kijpornyongpan and Aime (2017); This study
*Laurobasidium hachijoense* (syn. *Acaromyces ingoldii*) MCA4198	OQ516981	OQ540479	OQ516559	This study
*Meira miltonrushii* MCA3882^T^	JX432964	NR_120190	JX432962	Rush and Aime (2013)
*Mixia osmundae* (Outgroup) IFO 32048/IAM 14324^b^	D14163	DQ831010	DQ831009	Nishida et al. (1995); Matheny et al. (2006)
*Mycosarcoma maydis* (syn. *Ustilago maydis*) CBS 504.76/TKC58A^a^	KP322979	OQ540480	AF453938	Piepenbring et al. (2002); Wang et al. (2015); This study
*Pseudomicrostroma glucosiphilum* MCA4718^T^	KR912075	NR_148081	KR912072	Kijpornyongpan and Aime (2017)
*Testicularia cyperi* MCA3645	KU147241	KU147240	KU147242	This study
*Tilletiaria anomala* CBS 436.72^ T^	D83193	NR_111208	AY745715	Takashima and Nakase (1996); Matheny et al. (2006)
*Tilletiopsis washingtonensis* MCA4186	OQ516980	OQ540478	OQ516560	This study
*Violaceomyces palustris* SA807^T^	OQ516983	NR_136115	KM591583	Albu et al. (2015); This study

^T^indicates a holotype or an ex-type strain

^a^ KP322979 and AF453938 are sequences from CBS 504.76, and OQ540480 is a sequence from TKC58A

^b^ D14163 is a sequence from IFO 32048, and DQ831009 and DQ831010 are sequences from IAM 14324

*Representative ITS sequences of *Ceraceosorus* were used as references for both nucleotide variant discovery and phylogenetic analyses: OQ540416 and OQ540429 for *C. africanus*, OQ540463 for *C. americanus*, KT984947 for *C. bombacis*, and KT984939 for *C. guamensis*

To analyze the sequence variation of *Ceraceosorus* ITS regions, all sequenced ITS amplicons from all *Ceraceosorus* species were aligned in MEGA-X using the MUSCLE algorithm. The alignment was used for p-distance calculation using the MEGA-X built-in function, and for phylogenetic reconstruction using the Neighbor-Joining (NJ) method performed in MEGA-X with the Kimura-2 parameter as a nucleotide substitution model and 1000-replicate bootstrapping.

### Investigation of intragenomic variation

**Fig. 1:**
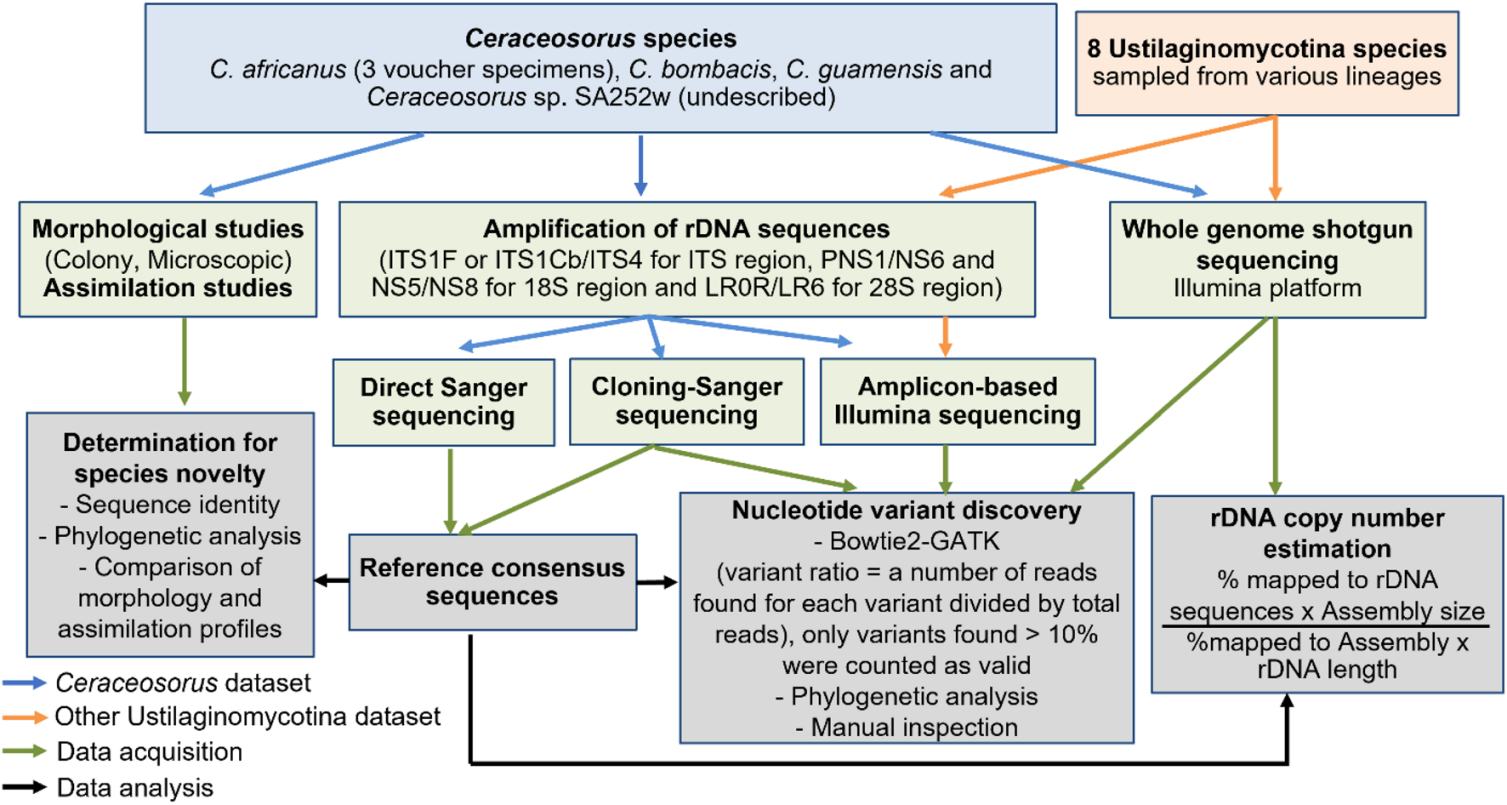
Analysis pipeline for this study

We detected intragenomic nucleotide variants within rDNA regions (partial 18S, partial 28S and ITS) from three data types—cloning-Sanger sequencing, targeted amplicon Illumina sequencing, and WGS Illumina sequencing (Fig. 1). For the ITS sequences of *Ceraceosorus* species obtained from PCR-cloning-Sanger sequencing, we aligned the ITS sequences of each species using the MUSCLE algorithm run in MEGA-X. The variant detection was done by visual inspection of the alignments. Any site having a variant site present in only one sequence for each strain/specimen was excluded from the valid variant list and we considered it as a PCR-cloning-sequencing error, unless the variant site was also present in other detection methods (see below). After the comparison with the other methods, the consensus nucleotide variants were used to correct PCR-cloning-sequencing errors from the *Ceraceosorus* ITS alignment. Correction was performed by manual exclusion of the nucleotide variant sites suspected as artefacts from the PCR-cloning-sequencing method (a variant present in one sampled clone and no consistent variant from other detection methods). The corrected ITS alignment was subjected to another p-distance calculation and phylogenetic reconstruction to evaluate the effect of the PCR-cloning-sequencing method on artefactual nucleotide variants.

The second method is targeted amplicon sequencing, which allows for sequence heterogeneity and provides greater coverage of sequenced regions. Amplification of rDNA regions was conducted as described above for the following species: all four *Ceraceosorus* species, *Me. miltonrushii*, *Tilletiopsis washingtonensis*, *My. maydis*, *P. glucosiphilum*, and *V. palustris*. PCR products from all rDNA regions for each species were pooled into a single sample and sent to the Purdue Genomics Core Facility for Illumina MiSeq sequencing. Library preparation and sequencing were performed using Illumina NexteraXT (Illumina, San Diego, CA, USA) and Illumina MiSeq 500 cycle kits, respectively. About 50,000 – 200,000 Illumina reads were generated for each sample. Adapter sequences and low-quality bases were trimmed using Cutadapt 1.12 (Martin 2011) and Trimmomatics 0.36 (Bolger et al. 2014). The cleaned Illumina MiSeq reads from each species were mapped to their reference rDNA sequences (Table 1) using Bowtie 2 2.3.3.1 (Langmead and Salzberg 2012). Bowtie 2 was run in the sensitive end-to-end mode, and other parameters were set as default. After that, we converted the sam alignment files to the bam format and added a read group for each single bam file using Samtools 1.6 (Li et al. 2009) and Picard 2.25.1 (http://broadinstitute.github.io/picard). Variant detection was then performed with GATK 3.8.0 (McKenna et al. 2010) using the HaploTypeCaller function with default parameters.

For WGS Illumina sequences, which provide greater coverage and avoid biases from amplicon-based approaches, raw sequencing reads from previously published WGS sequencing projects of several *Ustilaginomycotina* species were used for nucleotide variant detection: *C. guamensis*, *J. rosea*, *L. hachijoense* (syn. *A. ingoldii*),* Me. miltonrushii*, *P. glucosiphilum*, *Te. cyperi*, *Tilletiaria anomala*, *Tilletiopsis washingtonensis*, and *V. palustris* (Kijpornyongpan et al. 2018; Toome et al. 2014). Reads were retrieved from the sequencing read archive of the JGI MycoCosm genome portal (Grigoriev et al. 2014). For *C. bombacis*, the 300-bp paired-end WGS sequencing reads from a previous study (accession ERX541976, Sharma et al. 2015) were downloaded from the NCBI Sequence Read Archive database through the sra-toolkit. The WGS sequencing reads were assessed for read quality through FastQC 0.12.1 (Andrews 2010). We found the WGS sequencing reads from all datasets contain neither adapter sequences nor low-quality bases, so they were not subjected to further read cleaning. After that, the WGS sequencing reads from each species were mapped to their reference rDNA sequences using Bowtie 2 2.3.3.1 using the same setting for the targeted amplicon sequencing datatype, and subsequent variant detection was performed using the procedure described above.

For nucleotide variants from the vcf file generated by target-amplicon sequencing and WGS sequencing methods, any variant calls with read depth < 200, QD < 2.0, QUAL < 30.0, FS > 200.0 were manually discarded from the valid variant list. The average read depth of all called nucleotide variants across samples is approximately 2000 reads, we therefore used 10% of average read depth (200 reads) as a cutoff for discarding erroneous read mapping. Other values were minimal cutoffs from hard filtering for SNP and INDEL sites suggested by the GATK forum (https://gatk.broadinstitute.org/hc/en-us/articles/360035531112--How-to-Filter-variants-either-with-VQSR-or-by-hard-filtering). Exceptions are for the variants present in at least two nucleotide variant detection methods—they were not filtered out from the valid variant list. All other variant sites were also subject to compare with other detection methods to evaluate data consistency.

We also estimated the rDNA copy number of each genome by the following calculation: [a percentage of reads mapped to reference rDNA sequences x genome assembly size (in bp)] / [length of reference rDNA sequences (in bp) x a percentage of reads mapped to genome assembly]. This formula assumes a DNA read can be mapped once to a unique genomic region, while it can be mapped multiple times to rDNA repeats. As the rDNA repeats are not present in the reference genome assemblies of all studied species (Kijpornyongpan et al. 2018; Sharma et al. 2015; Toome et al. 2014), we compared a proportion of mapped reads between the genome assemblies and the reference rDNA sequences. The fold difference between proportion of mapped reads to the reference rDNA sequences and the genome assemblies was used as a proxy for rough estimation of rDNA copy numbers (Fig. 1).

Finally, the nucleotide variant lists from all three detection methods were compared to retrieve consensus nucleotide variant sites, as well as to evaluate consistency and conflict of called nucleotide variants. Due to resource and data availability, we noted that *C. bombacis* and *C. guamensis* are the only species for which nucleotide variant data were generated from all three detection methods. The other *Ceraceosorus* species lack WGS data and other *Ustilaginomycotina* species do not have data from the PCR-cloning-Sanger sequencing method. We calculated variant frequency for each nucleotide variant site as follows. For PCR-cloning-sequencing, a variant frequency equals the number of clones showing a variant site divided by the total number of clones. For targeted-amplicon sequencing and WGS mapping, a variant frequency equals the number of reads mapped to a variant site showing a variant base divided by the total number of reads mapped to the variant site. For samples having two or three independent runs of targeted-amplicon sequencing, we calculated average variant frequency and evaluated data consistency across independent runs (Table S2).

### Data visualization

All phylogenies were visualized in MEGA-X. We used the ggplot2 package (Wickham 2016), run in R 3.5.2 and RStudio 1.4.1103 to visualize detected nucleotide variants and variant frequencies in the *Ceraceosorus* ITS region. Other graphics were created and edited in Inkscape 0.92 (https://www.inkscape.org).

## Taxonomy

**Fig. 2:**
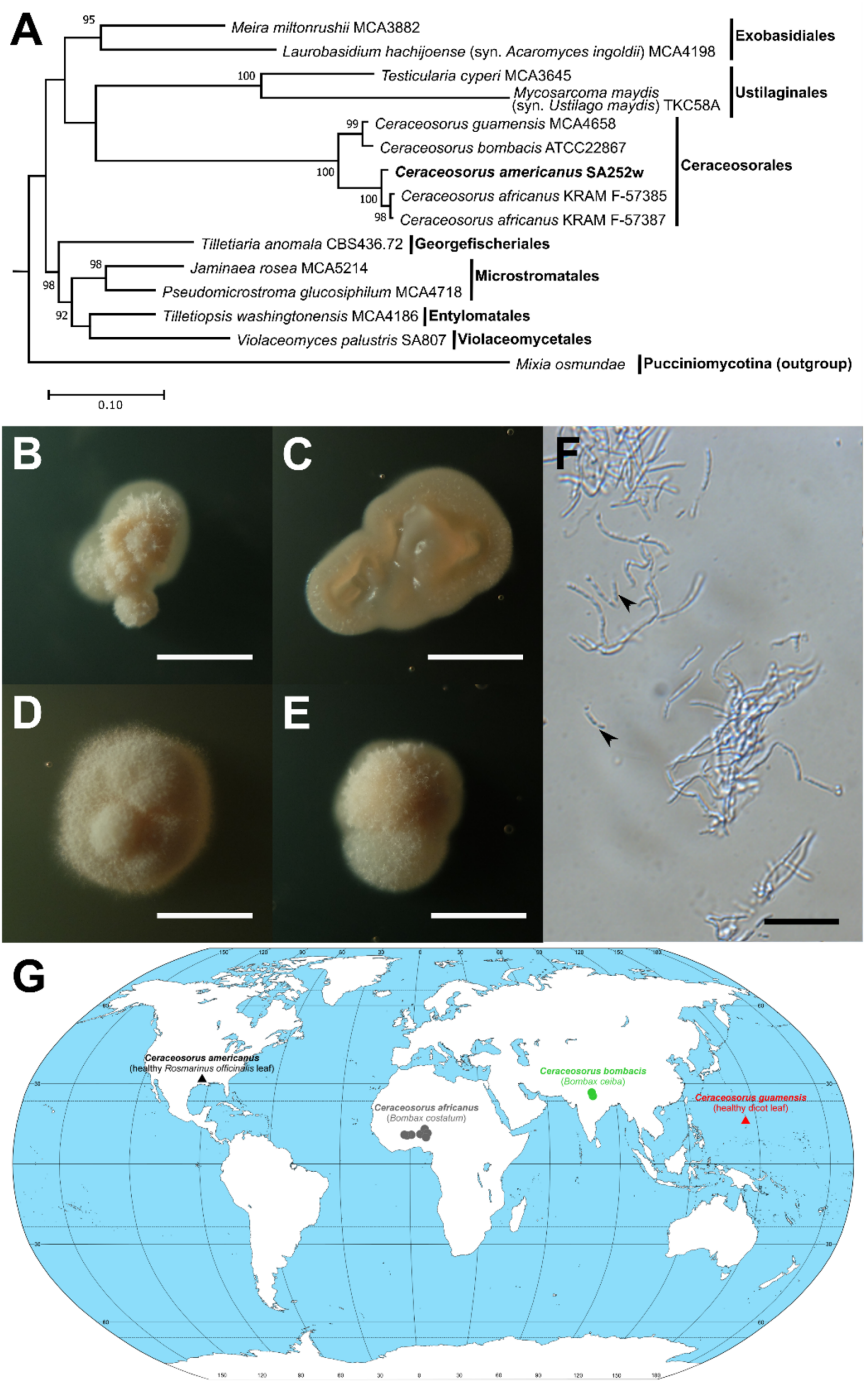
*Ceraceosorus americanus* sp. nov. (ex-holotype CBS144261 ≡ SA252w) **A** A phylogenetic tree confirming the placement *C. americanus* in *Ceraceosorales* (*Ustilaginomycotina*). The rDNA sequences (Table 1) of representative *Ustilaginomycotina* species and all described *Ceraceosorus* species, were aligned and concatenated. The alignment was used for phylogenetic reconstruction using RAxML with GTRCAT as the nucleotide substitution model and 1000-replicate bootstrapping. Bootstrap values are only shown for nodes with strong support (> 70%). *Mixia osmundae* was selected as an outgroup based on its placement in a different subphylum (*Pucciniomycotina*). **B–E** Colony morphology of ex-type CBS144261 on PDA (B), CMA (C), YMA (D), and YPGA (E). Bars: 2.5 mm. **F** Cell morphology in yeast malt broth (YMB) for 14 days. Cell suspension observed under a DIC microscope. Arrowheads indicate yeast-like cells. Bar: 20 μm. **G** Worldwide distribution of *Ceraceosorus* species. Sources of isolation are indicated in parentheses. Dots indicate phytopathogenic species with sexual stages. Triangles indicate species known only from asexual stages

*Ceraceosorus americanus* T. Kij. & Aime sp. nov. (Fig. 2, Table 2).

**Table 2:** Compounds that have different assimilation profiles among *Ceraceosorus* ex-type strains: ATCC 22867 for *Ceraceosorus bombacis*, MCA4658 for *Ceraceosorus guamensis*, and SA252w for *Ceraceosorus americanus*

Assimilation	ATCC 22867 C. bombacis*	MCA4658 C. guamensis*	SA252w *C. americanus*
*Carbon compound*			
Cellobiose	–	–	+
Lactose	–	–	+
D-Galactose	–	–	+
Palatinose	+	–	+
D-Melibiose	+	w	+
D-Arabinose	–	+	+
D-Ribose	w	+	+
Glycerol	–	+	+
D-Xylose	–	+	w
Gentiobiose	–	+	+
Ethanol	–	–	+
Maltitol	–	w	s
Fumaric acid	w	+	–
Xylitol	s	+	+
1,2-propanediol	–	–	+
Lactate	–	+	–
Methanol	w	–	+
*Nitrogen compound*			
Ethylamine-HCl	w	+	–

All *Ceraceosorus* strains can assimilate these compounds in common: D-glucose, sucrose, maltose, maltotriose, D-melezitose, D-raffinose, D-stachyose, L-arabinose, trehalose, turanose, D-mannitol, D-arabitol, *i*-erythritol, galactitol, succinic acid and soluble starch for carbon assimilation, and NaNO_2_ and KNO_3_ for nitrogen assimilation. They can also grow in vitamin-free medium, as well as in two osmotic media: 50% glucose yeast extract agar and 10% NaCl yeast extract agar. The maximum temperature all strains can grow in liquid culture is 30 °C. None of these strains assimilates these compounds: D-psicose, L-rhamnose, L-sorbose, α-methyl D-glucoside, D-galacturonate, D-glucuronate, methyl succinate, citrate, inositol, arbutin and salicin for carbon assimilation and L-lysine, cadaverine, creatine and creatinine for nitrogen assimilation

Sign: + , growth; –, no growth; w, weak growth; s, slow growth

*Assimilation data obtained from a previous study (Kijpornyongpan and Aime 2016)

MycoBank accession number: MB856345.

- Etymology: *americanus*, referring to the continent where the fungus was isolated, as this is the first species of *Ceraceosorus* found and collected in the Americas.
- Diagnosis: *Ceraceosorus americanus* differs from all other cultured *Ceraceosorus* species in its ability to assimilate cellobiose, lactose, D-galactose, and ethanol.
- Type: USA. Louisiana, East Baton Rouge Parish. Louisiana State University Campus, Baton Rouge. Isolated from the leaf surface of *Rosmarinus officinalis* (*Lamiaceae*) using the spore drop method. 17 February 2011, leg. S. Albu; PUL F22392, holotype as a dried culture; SA252w ≡ NRRL 66778 ≡ CBS 144261, ex-type cultures; GenBank accessions: KM591596, 18S ribosomal DNA sequence; KM591595, 28S ribosomal DNA sequence; OQ540449–OQ540477, ITS1-5.8S-ITS2 ribosomal DNA sequence.

Description: Colony morphology. After 14 days of incubation at room temperature (25 °C), colonies of *C. americanus* are as follows (Fig. 2B–E). Colonies on PDA are 2.5 mm in diameter, light cream (oac815), dull, velvety with filiform margins and elevated folds. Colonies on CMA are 3.5 mm in diameter, light cream (oac857), glabrous, butyrous with entire margins and flat elevations. Colonies on YMA are 4 mm in diameter, white cream (oac795), dull, velvety with filiform margins and elevated folds. Colonies on YPGA are 3 mm in diameter, light cream (oac814), dull, velvety with filiform margins and convex (dome) elevation. The average colony growth rate is 3.5 mm per 14 days, with the highest growth rate when cultured on YMA.

Micromorphology. After incubation in YMB with 200 round-per-minute shaking at room temperature (25 °C) for 7 days, the cell colony morphologies of *C. americanus* are as follows (Fig. 2F). Filamentous growth appears as branched, septate hyphae of 1.8–6.8 μm length × 1.1–1.7 μm diameter. Conidiospores not observed. Cells at a hyphal tip can detach from a hypha and behave yeast-like. Clamp connections absent. Sexual stage not observed.

Physiological properties. *Ceraceosorus americanus* can assimilate the following compounds as a carbon source: D-glucose, D-galactose, sucrose, cellobiose, lactose, maltose, maltotriose, D-melezitose, D-melibiose, D-raffinose, D-stachyose, D-ribose, D-arabinose, L-arabinose, D-xylose (weak), gentiobiose, palatinose, trehalose, turanose, D-mannitol, D-arabitol, *i-*erythritol, galactitol, maltitol (slow), xylitol, methanol, ethanol, 1,2-propanediol, succinic acid, and soluble starch. The fungus can assimilate potassium nitrate (KNO_3_) and sodium nitrite (NaNO_2_) as nitrogen sources. The following compounds are not assimilated by *C. americanus*: D-psicose, L-rhamnose, L-sorbose, α-methyl D-glucoside, D-galacturonate, D-glucuronate, methyl succinate, citrate, fumarate, lactate, inositol, arbutin, and salicin for carbon assimilation and ethylamine, L-lysine, cadaverine, creatine and creatinine for nitrogen assimilation. The fungus can grow in vitamin-free medium, as well as in two hyperosmotic media: 50% glucose yeast extract agar and 10% sodium chloride yeast extract agar. Fermentation is absent. The growth is positive at 30 °C, but negative at 35 °C.

Notes: *Ceraceosorus americanus* is the first *Ceraceosorus* species described from the Americas, while the other species are known from the Old World tropics: *C. africanus* from Benin, Ghana and Togo, *C. bombacis* from India, and *C. guamensis* from Guam (Fig. 2G). *Ceraceosorus africanus* and *C. bombacis* are known from the sexual morph as plant pathogens on the leaves of *Bombax costatum* and *B. ceiba*, respectively. Additionally, in the case of *Ceraceosorus bombacis* cultures were obtained in the laboratory, while for *C. africanus* culturing was not attempted. However, *C. americanus* and *C. guamensis* were both isolated from asymptomatic leaf phylloplanes and are only known from the asexual morph; it is unknown whether they also have a phytopathogenic strategy and sexual stages in their life cycle. The two phytopathogenic species live on *Bombax* hosts, which may indicate host specialization of the genus *Ceraceosorus* on this plant genus. In this case *C. americanus* and *C. guamensis* may have lost the phytopathogenic sexual stage because the potential hosts of the genus *Bombax* are absent in the Americas and Guam.

## RESULTS

### Analyses of intragenomic variation in the ITS regions of *Ceraceosorus*

First, we utilized the PCR-cloning-Sanger sequencing method to investigate sequence heterogeneity in the *Ceraceosorus* ITS region. A p-distance matrix was calculated from the cloned ITS sequences of all *Ceraceosorus* samples (Table S1). Maximum pairwise p-distances of ITS clones observed in each strain/specimen are as follows: 0.031 for *C. bombacis* ATCC 22867, 0.029 for *C. guamensis* MCA4658, 0.019 for *C. americanus* SA252w, 0.025 for *C. africanus* KRAM F-57386, and 0.029 for *C. africanus* KRAM F-57385 and KRAM F-57387. When all three specimens are considered, the maximum pairwise p-distance found in *C. africanus* is increased to 0.035, which exceeds the conventional 97% ITS identity cutoff used to separate operational taxonomic units in various studies (Tedersoo et al. 2015).

**Fig. 3:**
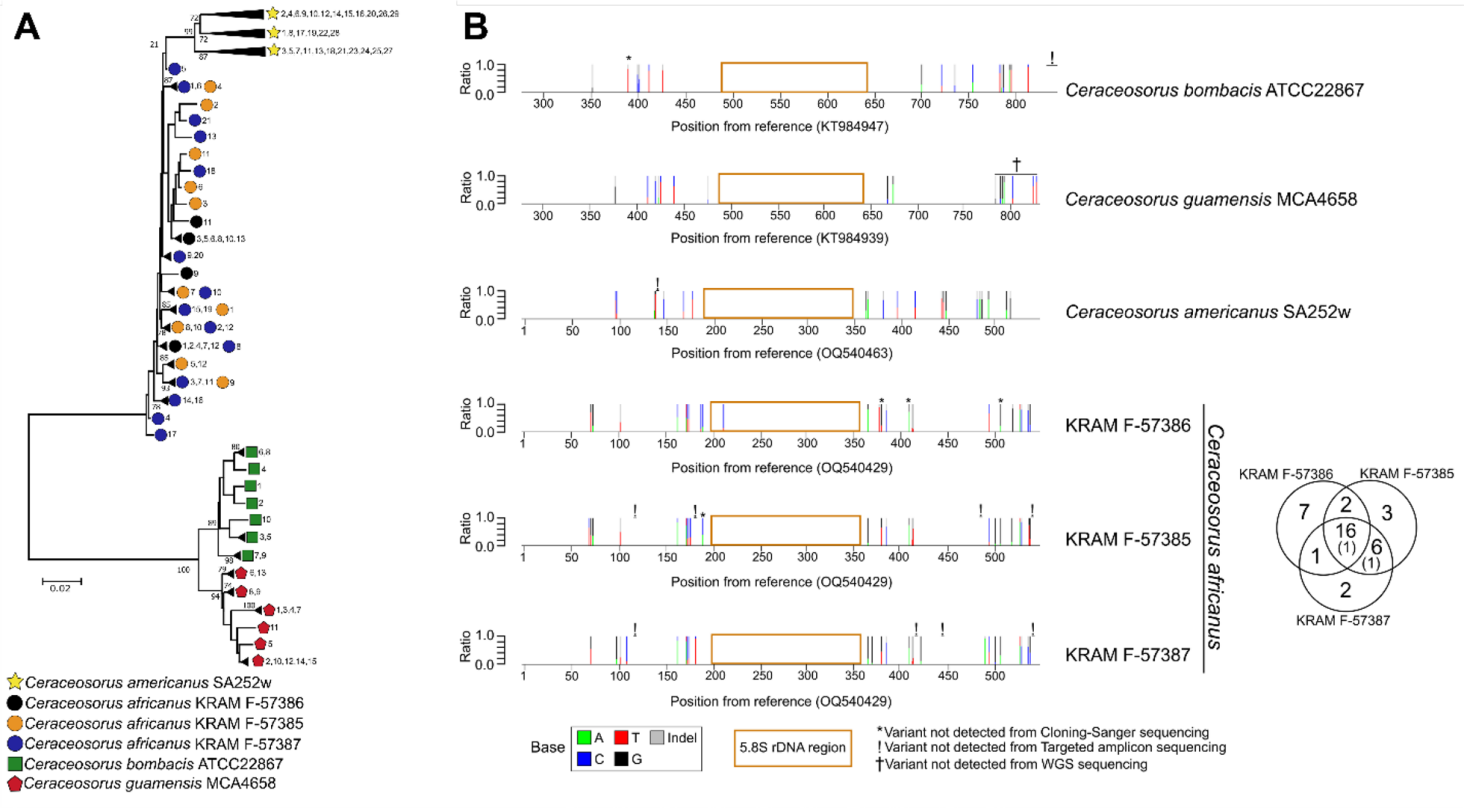
Intragenomic nucleotide variant detection of the internal transcribed spacer (ITS) regions among *Ceraceosorus* species. **A** The neighbor-joining tree of the ITS clones of all *Ceraceosorus* samples. The ITS amplicons from the PCR were cloned using T-vector and sequenced using the Sanger platform. The sequenced ITS regions were then aligned and used for neighbor-joining tree reconstruction. For better visualization, all sequences with branch lengths less than 0.01 substitutions/site are collapsed as a subtree. Only bootstrap values greater than 50 are shown on nodes. A symbol in each tip indicates a strain/specimen followed by clone number(s), which is in numerical order from a list of ITS clones (Table 1). A detailed phylogenetic tree can be found in Fig. S1. **B** Mapping of nucleotide variants in the ITS regions of all *Ceraceosorus* samples. The ITS amplicons from the PCR were used for targeted amplicon Illumina sequencing. Illumina raw reads were then mapped to the reference ITS sequences for nucleotide variant detection. For each stacked barplot, the X-axis is a position (in base pair) of the reference ITS sequence. The Y-axis is the ratio of nucleotide variants (A, T, C, G or indels) for each position. A position without a stacked line implies no heterogeneity (i.e., no intragenomic variation). Symbols over stacked lines indicate variants that are not detected in different discovery methods: cloning-Sanger sequencing, targeted amplicon sequencing, and WGS sequencing. A gold-framed rectangle on each plot indicates the 5.8S rDNA region of the reference sequence. A Venn diagram at the right side of *C. africanus* tabs indicates numbers of shared variant sites across three samples. Parentheses indicate numbers of shared variant sites in three samples but have conflicts in reference/variant nucleotides. Details of nucleotide variants can be found in Table S2

Phylogenetic reconstruction of *Ceraceosorus* ITS clones reveals that the intragenomic variation is intraspecific—no disparate ITS clone that overlaps across species is found (Figs. 3A, S1). We found variability among the three isolates of *C. africanus,* as the ITS clone clustering pattern is not random. The neighbor-joining tree indicates isolated placements of the KRAM F-57386 ITS clones compared to the ITS clones from the other two specimens. This correlates to the distant geographic location of KRAM F-57386 (collected from Benin) compared to KRAM F-57385 and KRAM F-57387 (collected from Ghana). The ITS clones of *C. africanus* KRAM F-57386 also have less intragenomic variation (average p-distance 0.012) than KRAM F-57385 (average p-distance 0.015) and KRAM F-57387 (average p-distance 0.014).

Next, we investigated intragenomic variation in the ITS region of all *Ceraceosorus* species through the targeted amplicon-Illumina sequencing approach. Our nucleotide variant detection showed that there are at least 15 variant sites in the ITS regions for each *Ceraceosorus* species—no variant site overlaps across different species (Fig. 3B, Tables 3, S2). For *C. africanus*, we see common variant sites in three specimens, and most common sites have consistent variant patterns (reference/variant base). However, KRAM F-57386 has more unique variant sites compared to the other two samples, 7 sites compared to 3 and 2 unique variant sites found in KRAM F-57385 and KRAM F-57387, respectively (Fig. 3B). One of the unique variant sites in KRAM F-57386 is in the 5.8S rDNA region. The reference ITS positions 500 and 537 are two common variant sites that have conflicting patterns..

**Table 3:** Summary of intragenomic nucleotide variants of *Ceraceosorus* species and other *Ustilaginomycotina* species detected by targeted-amplicon sequencing and whole genome shotgun (WGS) Illumina sequencing

Species Length of reference rDNA sequences (partial 18S, ITS, partial 28S)	Number of discovered variant sites in each rDNA region (substitutions/indels)						Estimated rDNA copy number from WGS data
	Targeted amplicon-Illumina sequencing^a^			WGS-Illumina sequencing			
	18S	ITS^b^	28S	18S	ITS	28S	
*Ceraceosorus bombacis* ATCC 22867 (1683, 846, 2165)	5/0 [2]	9/8 [3] (12/9 [10])	5/1 [2]	5/0	12/10	8/1	8
*Ceraceosorus guamensis* MCA4658 (1701, 849, 919)	3/1 [1]	11/5 [2] (12/5 [15])	9/0 [1]	3/1	6/4	9/0	7
*Ceraceosorus africanus* KRAM F-57386 (715, 592, 1054)	7/1 [2]	16/8 [2] (15/8 [13])	1/0 [2]	NA	NA	NA	NA
*Ceraceosorus africanus* KRAM F-57385 (715, 592, 1054)	3/1 [1]	18/7 [1] (20/6 [10])	0/0 [1]	NA	NA	NA	NA
*Ceraceosorus africanus* KRAM F-57387 (715, 592, 1054)	2/1 [1]	18/7 [1] (23/7 [20])	1/0 [1]	NA	NA	NA	NA
*Ceraceosorus americanus* SA252w (1052, 592, 1347)	3/1 [2]	13/8 [3] (13/8 [29])	1/0 [2]	NA	NA	NA	NA
*Meira miltonrushii* MCA3882 (1356, 614, 1076)	0/1^c^ [2]	0/0 [2]	0/0 [2]	0/0	0/0	0/0	14
*Pseudomicrostroma glucosiphilum* MCA4718 (1644, 688, 983)	0/0 [1]	1/0 [1]	0/0 [1]	0/0	1/0	0/0	17
*Tilletiopsis washingtonensis* MCA4186 (1720, 642, 1025)	1^c^/0 [2]	0/0 [2]	0/0 [2]	0/0	0/0	0/0	30
*Violaceomyces palustris* SA807 (1306, 676, 1037)	0/0 [1]	0/0 [1]	0/0 [1]	0/0	0/0	0/0	114
*Mycosarcoma maydis* (syn. *Ustilago maydis*) TKC58A (1954, 753, 1115)	0/0 [1]	0/0 [1]	0/0 [1]	NA	NA	NA	NA
*Laurobasidium hachijoense* (syn. *Acaromyces ingoldii*) MCA4198 (1713, 573, 1025)	NA	NA	NA	0/0	0/0	0/0	16
*Jaminaea rosea* MCA5214 (1618, 620, 1019)	NA	NA	NA	0/0	0/0	0/0	25
*Testicularia cyperi* MCA3645 (2003, 684, 1249)	NA	NA	NA	0/0	0/0	0/0	69
*Tilletiaria anomala* UBC591 (1749, 625, 1385)	NA	NA	NA	0/0	0/0	0/0	233

NA: not available, no data, analysis not conducted

^a^Square brackets indicate numbers of independent runs of targeted amplicon Illumina sequencing

^b^Parentheses indicate numbers of variant sites for ‘substitutions/indels [number of sampled clones]’ detected by cloning-Sanger sequencing method. Any variants present only in one sampled clone were not included here

^c^Variant sites are detected in one of two independent targeted amplicon sequencing runs but not in the WGS data. This can be an artefact due to errors in PCR-high throughput sequencing

We also detected ITS sequence heterogeneity in *C. bombacis* and *C. guamensis* based on WGS sequencing data, due to previous genome sequencing efforts (Kijpornyongpan et al. 2018; Sharma et al. 2015). Most of variant sites detected in the WGS sequencing data have consistent patterns to the results from the targeted amplicon sequencing method (Fig. 3B, Tables 3, S2). There are five variant sites in *C. bombacis* detected in the WGS data but not in the targeted amplicon data, while six variant sites in *C. guamensis* are detected in the targeted amplicon data but not the WGS data. From the targeted amplicon data and the WGS data, we noticed some observed nucleotide variant sites with only reads containing variant bases but not a reference base (Table S2). This indicates not only base homogeneity at those sites, but also inaccurate reference sequences used in the analyses. There is one site in the ITS sequence of *C. bombacis* ATCC 22867 (accession KT984947), two sites in the ITS sequence of *C. americanus* SA252w (accession OQ540463), and one site in the ITS sequence of *C. africanus* KRAM F-57385 (accession OQ540429) that were incorrectly sequenced.

We noticed conflicts of observed variant sites in three detection methods (Fig. 3B, Tables 4, S2). There are several variant sites that are detected by targeted amplicon Illumina sequencing but not by the PCR-cloning-Sanger sequencing—this is likely due to inadequate sampling efforts. Many observed variant sites from PCR-cloning-Sanger sequencing, with only one observed clone, are likely an artefact from PCR-cloning-Sanger sequencing, as they are not detected by targeted amplicon Illumina sequencing and WGS sequencing (Tables 4, S2). This is the most common source of conflict in variant calling. We also detected variant sites uniquely found in PCR-cloning-Sanger sequencing with two or more observed clones. Some of these sites are located in the 3’ end of ITS amplicons, making the nucleotide variants barely (or poorly) detectable in the targeted amplicon Illumina sequencing (Fig. 3B, Tables 4, S2). Some other sites are undetected, likely due to artefacts in the high-throughput sequencing, read mapping, or bioinformatic pipeline. In addition to undetected variants, the artefacts in data acquisition and data analyses can also lead to false variant calls in the targeted amplicon Illumina sequencing (Tables S2). We found approximately 5 – 25% of observed variant sites in each sample have inconsistent detection. Some of these, such as the positions 618, 620, 621 bp of *C. bombacis* ATCC22867 ITS region and the position 327 of *C. americanus* ITS region, can be identified as errors since the detected heterogeneity is found only in a single run of targeted amplicon sequencing but neither in other independent runs of targeted amplicon sequencing nor in WGS sequencing (Table S2). Incorrect variant calls can also result from inaccurate reference sequences, as described in a previous paragraph.

**Table 4:** Summary of conflicting nucleotide variants of *Ceraceosorus* ITS regions across different detection methods

Conflicting patterns	Possible source of conflict	Numbers of sites/cases with conflicting variant patterns*					
		ATCC 22867	MCA4658	SA252w^a^	KRAM F-57386^a^	KRAM F-57385^ab^	KRAM F-57387^ab^
Variant detected in Cloning-Sanger sequencing but not in other methods	Artefacts caused by PCR-cloning-Sanger sequencing	9	20	12	10	6	9
	3’ end of the amplicon, variant hardly detected in targeted amplicon sequencing	6	3	0	1	1	1
	Ambiguous to identify the source of conflict	0	0	0	2	3	4
Variant detected in targeted amplicon sequencing and WGS sequencing, but not in Cloning-Sanger sequencing	Inadequate clone sampling effort	1	0	0	3	1	0
Variant falsely called in targeted amplicon sequencing	Error in high-throughput sequencing and read mapping	3	0	1	0	0	0
Inconsistent variant calling among independent runs of targeted amplicon sequencing	Coverage inconsistency in high-throughput sequencing Error in high-throughput sequencing and read mapping	1	2	0	2	0	0
Only variant base detected, but not reference base	Inaccurate reference sequence	1	0	0	2	1	0
Others		0	4	0	0	3	0
Total		21	27	13	19	15	14

*Some cases/sites may have more than one conflicting pattern

^a^Samples do not have data from WGS sequencing

^b^Each sample has a single run of targeted amplicon sequencing

Finally, we re-analyzed the p-distance matrices and phylogenetic reconstruction of the cloned ITS sequences of *Ceraceosorus* after correcting PCR-cloning-sequencing errors. The maximum pairwise adjusted p-distances of ITS clones in each strain/specimen are as follows (Table S3): 0.025 for *C. bombacis* ATCC 22867, 0.019 for *C. guamensis* MCA4658, 0.023 for *C. americanus* SA252w, 0.017 for *C. africanus* KRAM F-57386, 0.025 for *C. africanus* KRAM F-57385, and 0.026 for *C. africanus* KRAM F-57387. The maximum adjusted pairwise p-distance found among all *C. africanus* specimens is 0.029, suggesting that the original p-distance is overestimated due to artefactual variant sites from PCR-cloning-sequencing errors. The phylogenetic tree reconstruction of the ITS after error correction reveals discrete haplotypes of ITS clones in each *Ceraceosorus* strain (Fig. S2). According to our data, there are at least 5 ITS haplotypes in *C. guamensis* MCA4658, 7 haplotypes in *C. bombacis* ATCC 22867 and *C. americanus* SA252w, 9 haplotypes in KRAM F-57386, 11 haplotypes in KRAM F-57385, and 15 haplotypes in KRAM F-57387. Taken together, these results suggest that the ITS regions of all *Ceraceosorus* samples do not evolve homogeneously.

### rDNA analyses from high-throughput sequencing data among *Ustilaginomycotina* species

We expanded our investigation on intragenomic rDNA variation to other *Ustilaginomycotina* species, as well as other rDNA regions in *Ceraceosorus* species (partial 18S and partial 28S). We sampled twelve fungal species, representing seven orders of *Ustilaginomycotina* (see Materials and Methods). Since we could use bulk Sanger sequencing to retrieve consensus sequences for the partial 18S and partial 28S regions of *Ceraceosorus* species and for all rDNA regions of other *Ustilaginomycotina* species, we decided to opt out the PCR-cloning-Sanger sequencing method for studies in these regions. The reason is that the intragenomic variation in these regions is expected to be low. Huge efforts of cloning-sequencing therefore are required to detect true signals of the sequence heterogeneity, while the high-throughput sequencing approach (including targeted amplicon and WGS data) allows the detection of minute signals. In addition, the PCR-cloning-sequencing method requires extra steps of molecular manipulation prior to sequencing. These include the replication of a plasmid containing an amplicon insert in *E. coli* and another colony PCR to obtain amplicons ready for sequencing. These extra steps may unnecessarily introduce more artefacts and that could interfere with true signals. Our empirical data from the *Ceraceosorus* ITS region showed several artefactual variant sites detected by the PCR-cloning-sequencing (Table S2). We therefore limited subsequent investigations to targeted amplicon sequencing and WGS sequencing.

Our study found *Ceraceosorus* as the only genus sampled with intragenomic variation in all rDNA regions (Table 3). The ITS region has the highest number of variant sites for all *Ceraceosorus* species. The partial 28S region has the lowest number of variant sites in *C. africanus* and *C. americanus*, while the lowest heterogeneity is found in the partial 18S region for *C. bombacis* and *C. guamensis*. The variant sites in the 18S and 28S regions detected by the WGS data of *C. bombacis* and *C. guamensis* match with most variant sites detected by the targeted amplicon sequencing, except that three additional substitution sites are detected only in the WGS data for the 28S region of C. bombacis (Tables 3, S2).

We performed targeted amplicon Illumina sequencing for five other *Ustilaginomycotina* species. Only three of which contain nucleotide variants in the rDNA regions that are not as highly variable as seen in *Ceraceosorus* (Tables 3). These species are as follows: *P. glucosiphilum* MCA4718 with one substitution site in the ITS region, *Me. miltonrushii* MCA3882 with one indel site in the partial 18S region, and *Tilletiopsis washingtonensis* MCA4186 with one substitution site in the partial 18S region. We also examined rDNA sequence heterogeneity using the WGS data from eight *Ustilaginomycotina* species, four of them were previously examined through the targeted amplicon sequencing method. *Pseudomicrostroma glucosiphilum* MCA4718 is the only species that has a nucleotide variant site from the WGS data. The variant site is a substitution in the ITS region, which is the same variant site detected in the targeted amplicon sequencing method (Tables 3, S2). However, the WGS data of *Tilletiopsis washingtonensis* MCA4186 and *Me. miltonrushii* MCA3882 did not find variant sites previously detected in the targeted amplicon sequencing data (Tables 3, S2). This can be due to inconsistency across multiple high-throughput sequencing runs, or errors/artefacts occurred during PCR and sample preparation prior high-throughput sequencing.

Although intragenomic variation in rDNA sequences is absent in most of the *Ustilaginomycotina* species analyzed in this study, we noticed several nucleotide variants that are falsely called by bioinformatic analyses (Table S2). There are two main reasons for false calling. The first reason is inaccurate reference sequences, even from the same fungal strains/isolates used for targeted amplicon Illumina sequencing and/or WGS Illumina sequencing. This occurs in the ITS and partial 18S regions of *C. guamensis* MCA4658 (accessions: KT984939 and KT984925), the ITS and partial 28S regions of *Me. miltonrushii* MCA3882 (accessions NR120190 and JX432962), the partial 18S region of *Tilletiaria anomala* UBC591 (accession D83193), and the ITS region of *Tilletiopsis washingtonensis* MCA4186 (accession OQ540478, which was sequenced in our study). The second reason comes from potential errors/artefacts introduced during PCR and/or sample preparation for high-throughput sequencing. Examples are the variant sites detected in *Tilletiopsis washingtonensis* MCA4186 and *Me. miltonrushii* MCA3882 as described above. These false calls are a warning that nucleotide variants retrieved from the bioinformatic pipeline need to be carefully examined and interpreted.

Finally, we estimated rDNA copy numbers in all *Ustilaginomycotina* species for which WGS data and reference genomes are available. Based on our calculation, the rDNA copy number ranged from 7 to 233 copies with a median of 20.5 (Table 3). The two *Ceraceosorus* species for which genomic data are available have the lowest copy numbers: 7 copies for *C. guamensis* and 8 copies for *C. bombacis*. Two species with the highest estimated rDNA copy number are *Tilletiaria anomala* UBC591 (233 copies) and *V. palustris* SA807 (114 copies).

## DISCUSSION

### Geographical distribution and ecological role of *Ceraceosorus*

*Ceraceosorus americanus* represents the first report of *Ceraceosorus* from the Western Hemisphere. The other species of the genus have been discovered in distant locations in subtropical zones: *C. africanus* in West Africa, *C. bombacis* in India, and *C. guamensis* in Guam (Cunningham et al. 1976; Kijpornyongpan and Aime 2016; Piątek et al. 2016) (Fig. 2G). *Ceraceosorus bombacis* and *C. africanus* are plant pathogenic fungi on *Bombax* spp. leaves, while *C. guamensis* and *C. americanus* are thus far known only as asymptomatic epiphytic fungi on plant leaves. A recent metagenomic study found the DNA sequence of *Ceraceosorus* sp. on the surface of citrus fruits collected in China, and its network analysis suggested the role of *Ceraceosorus* sp. in maintaining the stability of a fungal community structure (Jing et al. 2023). Ecological data on *Ceraceosorus* species are almost entirely lacking, probably due to a combination of factors. The extremely slow growth rate in culture (1–2 mm in diameter per week) limits isolation by conventional methods from environmental sampling (Kijpornyongpan and Aime 2016). Additionally, the ITS region in studied *Ceraceosorus* species contains multiple mismatches at the annealing region of most commonly used fungal DNA barcoding primers, making it likely that environmental DNA studies may fail to record its presence (Kijpornyongpan and Aime 2016).

### Prevalence of intragenomic rDNA variation in fungi

Our previous work shows high intragenomic variation of the ITS region in *C. bombacis* and *C. guamensis* (Kijpornyongpan and Aime 2016). For this study, we expanded our investigation in terms of examined rDNA regions (partial 18S, ITS, and 28S) and taxonomic range (two additional *Ceraceosorus* species, plus representative ‘smut’ fungi from 7 orders of *Ustilaginomycotina*). As the first subphylum-wide survey of intragenomic rDNA variation in *Ustilaginomycotina*, we found that only species in the genus *Ceraceosorus* (*Ceraceosorales*) have a high degree of ITS sequence heterogeneity, such that obtaining consensus sequences from direct Sanger sequencing usually fails (Kijpornyongpan and Aime 2016; Piątek et al. 2016). We acknowledge that our study had a limited number of samples for the genus *Ceraceosorus* as well as for the subphylum *Ustilaginomycotina*, and that may not fully represent the total intragenomic variation existing in these fungi. One reason is because the genus *Ceraceosorus* is one of the most overlooked genera in the subphylum. In addition, scarce sampling and sequencing efforts in *Ustilaginomycotina* result in underrepresentation of genomic data for the analyses. Despite these limitations, our study shows that extensive rDNA intragenomic variation is explicitly found in *Ceraceosorus*.

The failure to directly sequence the ITS region by Sanger methods without ambiguous bases also occurs in several fungi (Alper et al. 2011; Connell et al. 2010; Harrington et al. 2014; Liu et al. 2015; Pringle et al. 2000; Roscini et al. 2018; Wang and Yao 2005; Woo et al. 2010; Zhao et al. 2015). In-depth investigations of many studies also show that intragenomic variation of the rDNA repeats exists in several fungal lineages including *Agaricomycotina* (Cruz et al. 2014; Ko and Jung 2002; Lindner et al. 2013; Lindner and Banik 2011; Smith et al. 2007; Vydryakova et al. 2012; Wang and Yao 2005), *Glomeromycotina* (de Souza et al. 2018; Lin et al. 2014; Redecker et al. 1999; Thiéry et al. 2016, 2012), Mucoromycota (Woo et al. 2010), *Pezizomycotina* (Bradshaw et al. 2023; Kovács et al. 2011; Li et al. 2017, 2013; Naidoo et al. 2013; Poczai et al. 2015; Rooney and Ward 2005; Stadler et al. 2020), *Pucciniomycotina* (Moricca et al. 1996, McTaggart and Aime 2018), and *Saccharomycotina* (Colabella et al. 2021; Dakal et al. 2016; James et al. 2009; Sipiczki et al. 2018).

From these studies, we present three aspects of intragenomic variation that need to be considered: (1) variant sites (number of variant sites, from one to dozens of sites; types of variant sites, e.g., substitutions, indels or long insertions/deletions), (2) haplotypes (number of haplotypes, from two to twenty haplotypes; *p*-distance, from < 1% to 20% differences), and (3) frequencies of each variant site/haplotype in the genome (from one count of clone/sequencing read up to 50% frequency). Various studies report different aspects of intragenomic variation, depending on their methodology and data presentation. Most studies referenced in the previous paragraph report haplotype variation (2), with the most popular type of analyses conducted as phylogenetic trees, and occasionally as network analyses (Dakal et al. 2016; Sipiczki et al. 2018) and p-distance matrices (Cruz et al. 2014; Li et al. 2017; Smith et al. 2007). Fewer studies present data on variant sites (1) by displaying sequence alignments (Alper et al. 2011; Harrington et al. 2014; Ko and Jung 2002; Liu et al. 2015; Moricca et al. 1996; Redecker et al. 1999; Wang and Yao 2005; Woo et al. 2010), depicting diagrams showing variant sites along rDNA regions (Colabella et al. 2021; James et al. 2009; Li et al. 2017; Lin et al. 2014; Lindner and Banik 2011; Poczai et al. 2015; Roscini et al. 2018; Stadler et al. 2020; Vydryakova et al. 2012), or tabulating the number of substitution/indel sites (Dakal et al. 2016; Smith et al. 2007). The third aspect (variant frequency) has the least attention in the literature (Harrington et al. 2014; James et al. 2009; Lin et al. 2014; Lindner and Banik 2011; Roscini et al. 2018; Sipiczki et al. 2018), although the proportion of nucleotide polymorphisms in a genome is informative for understanding the underlying mechanisms for sequence heterogeneity. A few other studies incorporate other molecular aspects of rDNA sequence heterogeneity such as calculating sequence evolutionary rates (Li et al. 2017, 2013; Poczai et al. 2015; Thiéry et al. 2016) and examining the effect of nucleotide polymorphisms on rRNA secondary structures (Li et al. 2017, 2013; Poczai et al. 2015; Sipiczki et al. 2018; Stadler et al. 2020). Future integration of all aspects of intragenomic rDNA variation in multiple fungi across the kingdom will shed light on the molecular mechanisms for this phenomenon.

### rDNA sequence heterogeneity and concerted evolution

Concerted evolution theory says that gene homogenization occurs through DNA recombination among rDNA repeats, and that this mechanism can repair or remove variants from the consensus sequences of the rDNA repeats (Eickbush and Eickbush 2007; Liao 1999). Because the rDNA repeat in *Ceraceosorus* species appears to have partially escaped from concerted evolution, we conducted additional analyses to search for potential mechanisms that diminish gene homogenization, using genomes generated from previous studies. These are from two *Ceraceosorus* species—*C. bombacis* and *C. guamensis* (Kijpornyongpan et al. 2018; Sharma et al. 2015) and seven other representatives of *Ustilaginomycotina* (Kijpornyongpan et al. 2018), which appear to fit the concerted evolution model due to their homogenous rDNA sequences. The genomes of the two *Ceraceosorus* species have the lowest estimated rDNA copy number of all *Ustilaginomycotina* species analyzed (Table 3). Under the gene homogenization model, we postulate that homogenization becomes difficult in genomes with lower copy numbers of rDNA repeats. West et al. (2014) found an association between rDNA repeat types in *Saccharomyces cerevisiae* genomes (homogeneous/mosaic) and rDNA copy number. However, analyses of data from *Hypoxylaceae* species showed no association between rDNA copy number and the number of variant sites (Stadler et al. 2020). Loss of molecular machinery in DNA recombination and repair is another probable mechanism that impedes gene homogenization (Kobayashi and Sasaki 2017; Liao 1999; Paloi et al. 2022). Through comparative genomic analyses in the JGI MycoCosm database, we examined genes that are absent in the *C. guamensis* genome but present in other *Ustilaginomycotina* genomes. Although we could not attribute any of these missing genes to DNA recombination and repair (Fig. S3), further investigation of mutated genes (Kobayashi and Sasaki 2017) in *Ceraceosorus* genomes is promising for a better understanding of an underlying mechanism of concerted evolution.

Although recent studies suggest that intragenomic variation of rDNA regions is widespread in fungi (Bradshaw et al. 2023; Paloi et al. 2022; Stadler et al. 2020), concerted evolution generally maintains sequence homogeneity of rDNA regions. We propose several ways in which rDNA sequences may escape concerted evolution and propagate intragenomic variation. Hybridization is the most common source of rDNA sequence heterogeneity, often found in fungi that are predominantly diploid or dikaryotic (Dakal et al. 2016; Huang et al. 2010; Hughes et al. 2013; James et al. 2009; Ko and Jung 2002; Moricca et al. 1996; Naidoo et al. 2013; Paloi et al. 2022; Sipiczki et al. 2018; Tremble et al. 2020; Vydryakova et al. 2012; Wang and Yao 2005; West et al. 2014; Woo et al. 2010). Naidoo et al. (2013) demonstrated that these heterogeneous rDNA copies can gradually become homogenized after a few generations of sexual reproduction, a process that involves meiosis, crossing-over, and DNA recombination. However, the data from Dakal et al. (2016) suggest that partial homogenization can occur, which creates more variability in different parts of the rDNA array. Hybridization and partial gene homogenization consequently lead to gene genealogy discordance and the emergence of ‘species complexes’, which are problematic for fungal species identification (Paloi et al. 2022).

The second source of sequence heterogeneity comes from accumulated mutations (Cruz et al. 2014; Kijpornyongpan and Aime 2016; Li et al. 2017, 2013; Lin et al. 2014; Lindner et al. 2013; Lindner and Banik 2011; Poczai et al. 2015; Rooney and Ward 2005; Wang and Yao 2005). Mutant rDNA copies can avoid conversion to the original copy (i.e., relaxed concerted evolution) through prolonged asexuality (Lin et al. 2014), ineffective DNA recombination and repair (Kobayashi and Sasaki 2017), or conversion to pseudogenes (Li et al. 2017, 2013; Poczai et al. 2015; Sipiczki et al. 2018; Stadler et al. 2020). While subtle rDNA sequence heterogeneity (> 97% similarity) is not a problem for species identification, the emergence of pseudogenes may be, as these may have much lower similarity (ca. 90%) compared to consensus rDNA sequences (Stadler et al. 2020).

**Fig. 4:**
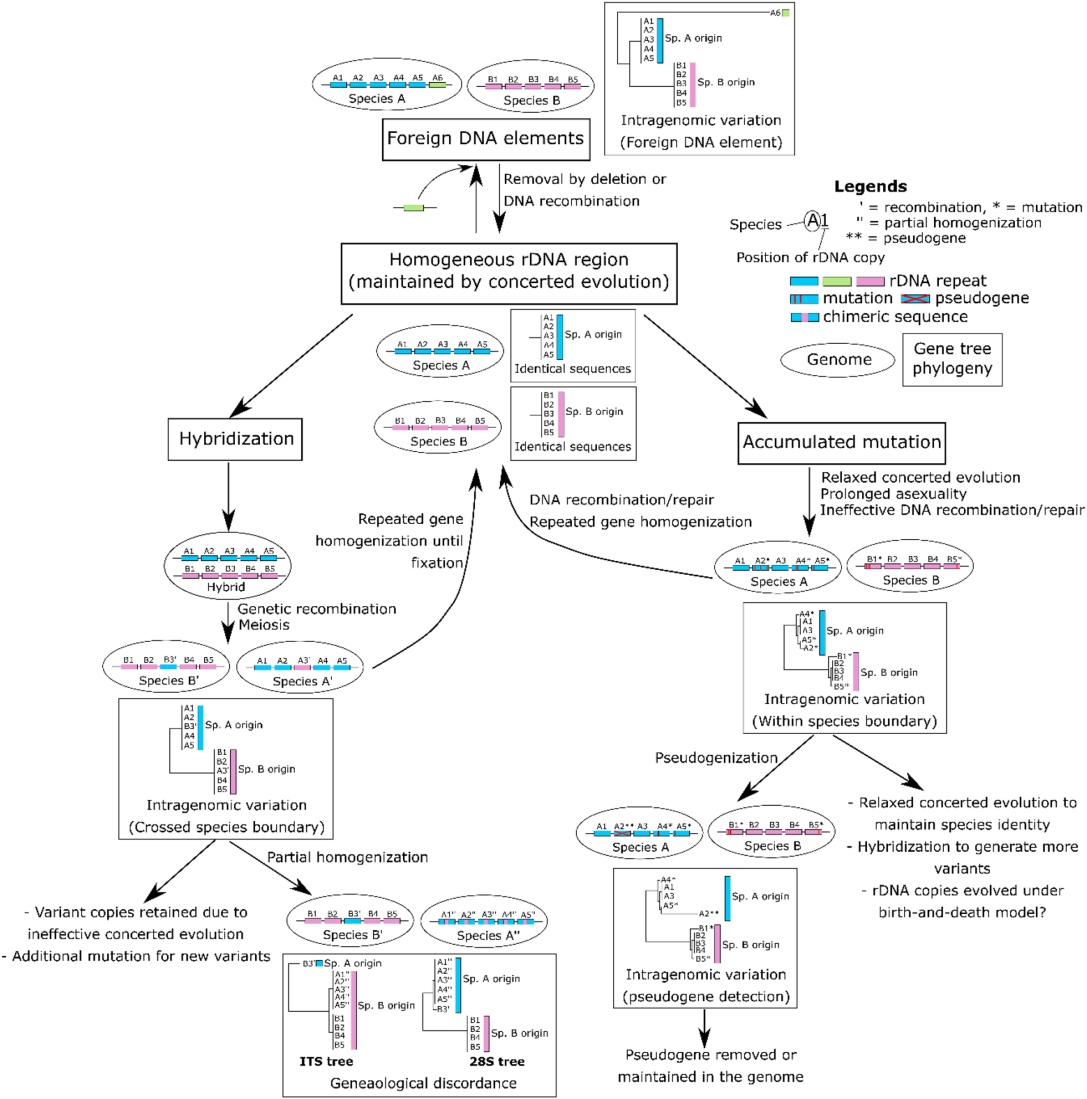
Schematic illustration of possible sources for intragenomic rDNA variation. The model assumes the homogeneity of rDNA sequences by default. We propose three major sources of sequence heterogeneity as follows: hybridization, accumulated mutation, and foreign DNA elements. The rDNA copies in each species/genome are shown in ovals. An alphabet followed by a number indicates the ID for each rDNA copy. Graphs in rectangles illustrate how gene trees look like in each scenario. Branch lengths are proportional to phylogenetic distances. Gene duplications, deletions, and translocations are not shown here to simplify the model

The final source of sequence heterogeneity is the introduction of foreign elements, such as group I introns (Bradshaw et al. 2020) or rDNA sequences from distantly related species (Redecker et al. 1999; Virtudazo et al. 2001). These foreign elements can cause a problem not only in species identification, but also in the inaccuracy of genome assembly (Bradshaw et al. 2023). Based on p-distance and ITS gene phylogeny, the observed intragenomic variation of *Ceraceosorus* is consistent with an interpretation of accumulated mutation under relaxed concerted evolution, since all ITS haplotypes of each species do not cross species boundaries (Figs. 3 and S2, Table S3). The higher degree of sequence heterogeneity in the ITS region, compared to the coding regions 18S and 28S, also supports that the rDNA regions were subject to mutation-selection scenario. Our proposed model for the emergence of intragenomic variation in rDNA is summarized in Fig. 4.

### Assessment of methods used in detecting intragenomic rDNA variation

Currently, there are several sequence-based methods for detecting intragenomic variation of the rDNA region in fungi: PCR-direct Sanger sequencing, PCR-cloning-Sanger sequencing, targeted amplicon high-throughput sequencing, WGS high-throughput sequencing, and genome data mining (Paloi et al. 2022). Each method has advantages and disadvantages, which are summarized in Table 5.

**Table 5:** Summarized properties of various detection methods for studying intragenomic variation

Variant detection method Properties	Direct Sanger sequencing	PCR-cloning-Sanger sequencing	Targeted amplicon high-throughput sequencing	Whole genome shotgun (WGS) high-throughput sequencing	Data mining from genome assembly
Procedure to detect intragenomic variation	Directly use PCR amplicons for Sanger sequencing	- Sample clones from PCR amplicons prior Sanger sequencing - Make an alignment of cloned sequences to examine sequence heterogeneity	- Use PCR amplicons for library preparation for high-throughput sequencing (Illumina, Ion Torrent or similar methods) - Make an alignment or perform read mapping (Bowtie2, BWA, BLAST) against reference sequences and then nucleotide variant calling (e.g. GATK)	- Use genomic DNA for library preparation for high-throughput sequencing or long-range sequencing (PacBio, Nanopore) - Make an alignment or perform read mapping (Bowtie2, BWA, BLAST) against reference sequences and then nucleotide variant calling (e.g. GATK)	- Use reference sequences as a seed to locate each rDNA copy from a genome assembly - Retrieve sequences from all copies and make an alignment to examine sequence heterogeneity
Signs that indicate intragenomic variation	- Failure to obtain consensus rDNA sequences - Overlapping peaks from chromatogram	Substitutions/indels from the alignment	- Nucleotide difference from reference sequences - Substitutions/indels from the alignment	- Nucleotide difference from reference sequences - Substitutions/indels from the alignment	Substitutions/indels from the alignment
Types of information obtained -Variant site	- Able to indicate few substitutions from chromatogram	- Yes	- Yes	- Yes	- Yes
- Haplotype	- No	- Yes	- Limited to short amplicons like ITS1 and ITS2	- Limited to short amplicons like ITS1 and ITS2 for short read sequencing, no limitation in long-range sequencing	- Yes
- Frequency (like pSNPs, haplotype frequency)	- No	- Limited to a number of sampled clones	- Yes	- Yes, also possible to estimate rDNA copy number	- Yes, also possible to estimate rDNA copy number
Advantages	First observational data to suspect intragenomic variation without extra procedures	- Traditional method that represent all aspects of intragenomic variation - Able to detect complex variants or low-coverage regions that cannot be detected in high-throughput sequencing	- High coverage, abundant information about variant sites and pSNPs - Not labor intensive	- High coverage, abundant information about variant sites and pSNPs - Able to retrieve haplotype data and rDNA array architecture if using long-range sequencing - Not labor intensive	- Able to retrieve all aspects of intragenomic variation, as well as rDNA array architecture - Sequencing not required
Disadvantages	- Unable to provide details on sequence heterogeneity (variant site, haplotype, frequency) - Unable to detect intragenomic variation when variant haplotype is present at low frequency	- Unable to detect variants not captured by PCR, PCR bias - Data completeness depending on laborious cloning efforts - Potential overestimation of variants caused by artefacts/errors from PCR-cloning-sequencing	- Unable to detect variants not captured by PCR, PCR bias - Inconsistency of data acquisition, which affects detected variant sites and pSNPs - Errors/artefacts in variant calling, probably caused by amplification error, bioinformatic pipeline or improper reference data	- Most costly compared to other methods - Uncontrollable low sequencing coverage regions - Errors/artefacts in variant calling, depending on bioinformatic pipeline and reference data	- Repetitive elements, like rDNA sequences, frequently ignored and discarded in genome assemblies - Less data completeness compared to utilizing raw sequencing data - Insufficient rDNA copies in the assemblies for the analyses
Recommendation	Require additional sequencing methods	- Provide a screening method to eliminate errors/artefacts, e.g., removing variant sites observed from a single clone - Sample at least 15 clones per specimen for good coverage (according to our study) - Avoid clone oversampling to reduce erroneous variants	- Have at least two independent sequencing runs to check data consistency, report only consensus variants that are present in most runs - Set a read number/frequency cutoff for reporting valid variant sites (e.g., discard variant sites that have representing reads less than 10% of total read numbers) - Clearly report detailed bioinformatic pipeline	- Set a read number/frequency cutoff for reporting valid variant sites (e.g., discard variant sites that have representing reads less than 10% of total read numbers) - Clearly report detailed bioinformatic pipeline	- Ensure the genome assembly quality and presence of multiple rDNA copies prior to analyses - Cross-check with raw sequencing data to assure equivalent rDNA copy number estimation and to prevent data loss

PCR-cloning-Sanger sequencing is a traditional method that can provide information about variant sites, haplotypes, p-distance, and frequencies of variant sites/haplotypes (depending on the number of clones). However, this method is the largest source of artefacts, which are not found in sequences generated by the other methods (Tables 4, S2). Pairwise p-distance values are also significantly reduced after data correction by discarding variant sites found in only one clone but not in the other detection methods (Tables S1, S3). Few previous studies have attempted to deal with PCR errors, such as using high fidelity DNA polymerase (Kovács et al. 2011; Poczai et al. 2015), performing parallel PCR (Kovács et al. 2011), and testing secondary clones to check for *Taq* DNA polymerase misreading (Simon and Weiß 2008). However, errors can occur at any stage where a nucleotide base is incorporated into a new strand of DNA. This includes not only initial PCR amplification, but also multiplication of a cloned vector during bacterial colony growth, secondary colony PCR to obtain a desired amplicon, and the dideoxy-chain termination process during Sanger sequencing. Given this, there is a possibility that several previous studies may have overestimated variant sites, haplotypes and/or p-distances, especially those that are represented from one sampled clone (Alper et al. 2011; Ganley and Kobayashi 2007; Harrington et al. 2014; Liu et al. 2015; Pannecoucque and Hofte 2009; Simon and Weiß, 2008; Thiéry et al. 2012; Vydryakova et al. 2012). A variant present in a single clone is not always an artefact to be discarded. What we suggest is that intragenomic variants need to be carefully examined, and comparison with other detection methods is one of possible solutions.

In contrast to the PCR-cloning-Sanger sequencing method, high-throughput sequencing requires less steps for data acquisition. The targeted amplicon sequencing requires fewer rounds of PCR amplification of the desired rDNA region prior to library construction and sequencing, whereas WGS sequencing does not require PCR amplification. These two methods therefore minimize potential PCR-related artefacts/errors. Our analyses on various *Ustilaginomycotina* non-*Ceraceosorus* species show that most of the studied rDNA regions do not have sequence heterogeneity (Table 3), except one heterogeneous site in the ITS region of *P. glucosiphilum* (Tables 3, S2). A detected heterogeneous site in the partial 18S region of *Tilletiopsis washingtonensis* is likely due to an artefact as the detected variant site is only found in one of two independent runs for the targeted amplicon sequencing but not found in the WGS data (Table S2). In contrast, it cannot be determined whether a detected heterogeneous site in the partial 18S region of *Me. miltonrushii* is an artefact or not since it was detected in two independent runs of the target amplicon sequencing but not in the WGS data. In our analyses of ITS regions in each *Ceraceosorus* sample, we observed up to five variant sites that have inconsistent patterns across independent runs from targeted amplicon sequencing (Table 4): some can be identified as errors in amplification/sequencing/read mapping while others are hard to determine. Despite this ambiguity, the chance of erroneous variant sites by the targeted amplicon sequencing is less than from PCR-cloning-Sanger sequencing (Tables 4, S2).

High-throughput sequencing, such as Illumina and Ion Torrent sequencing, is a robust method for generating high sequencing depth and coverage, which allows the detection of rare variant sites not detected by cloning-Sanger sequencing (Tables 4, S2; Lindner et al. 2013; Colabella et al. 2021). This method also allows for the genome-scale study of variant frequencies, termed as ‘partial single nucleotide polymorphism (pSNP)’ (James et al. 2009; West et al. 2014). However, there are a few limitations to this method that need to be carefully considered. First, there are two approaches for performing high-throughput sequencing: the targeted amplicon approach and the WGS approach. The former approach may introduce PCR-bias (Tedersoo et al. 2015), especially if rDNA sequences, even functional regions or pseudogenes, have evolved to where they cannot be annealed to by common PCR primers (Kijpornyongpan and Aime 2016; Li et al. 2017, 2013). Although the latter approach can capture sequences that may be missed during PCR amplification (Lin et al. 2014; this study), it requires high sequencing depth and additional DNA parsing to retrieve sequences of interest. The second limitation comes from data collection. We notice inconsistency in sequence coverage between independent runs of targeted amplicon Illumina sequencing within the same sample. This affects the detection of some variant sites, as well as the frequencies of many detected variant sites (Tables 4, S2). The sequencing inconsistency is also found in 454 pyrosequencing (Lücking et al. 2014) and WGS Illumina sequencing (Lin et al. 2014). The third limitation comes from data analysis. Incorrect read mapping, indicated by low coverage (< 10% of average read depth, which is 200 reads in this study), can lead to incorrect variant calls (Tables 4, S2). Stringent read mapping software such as Bowtie2 and BWA may not detect sequencing reads that are too divergent from reference sequences, such as rRNA pseudogenes (Li et al. 2017). Finally, there is limited capacity to perform p-distance calculation and haplotype analysis data from short sequencing reads on longer regions such as the 18S and 28S rDNA.

Data mining from genome assembly is a discovery method that can provide information on variant sites, haplotypes, p-distances, frequencies of variant sites/haplotypes, and estimated rDNA copy number (Bradshaw et al. 2023; Li et al. 2017; Paloi et al. 2022; Stadler et al. 2020). Despite the potential to analyze all aspects of intragenomic variation within a single platform, the use of genome assembly data requires special precautions. The rDNA region is a part of repetitive elements that are often discarded from the final DNA assembly constructed from short read high-throughput sequencing (Treangen and Salzberg 2012). Thus, in many genome assemblies, the rDNA region is absent or present at much lower copy numbers than in intact genomes in the organisms. A recent study by Bradshaw et al. (2023) reveals that 2251 out of 2414 genomes analyzed have ITS copy numbers < 10, and 694 of them have no ITS sequences present in the assembly. However, Lofgren et al. (2019) estimated that the rDNA copy number of fungal species analyzed in their study ranges from 14 to 1,442 copies. Although genome assemblies constructed from long-ranged sequencing tend to have more ITS copy numbers than genome assemblies constructed from short read sequencing, data from Bradshaw et al. (2023) revealed that about 50% of reference genomes generated from PacBio/Nanopore sequencing (235/476 genomes) have less than 5 copies of ITS region in the DNA assembly. Therefore, analyses from raw sequencing data will provide more completeness by preventing information from being discarded during DNA assembly.

Considering our results and those of other studies, we provide some recommendations for future research on intragenomic variation of rDNA, as well as similar multigene families. First, having at least two independent runs (i.e., technical replicates) for each type of sequencing is a good approach to avoid inconsistencies in downstream analyses. This is not only to address data inconsistencies from high-throughput sequencing, but also to ensure the accuracy of reference sequences used for nucleotide variant discovery. Examples are our detection of inaccurate rDNA sequences used as references for *C. bombacis* ATCC22867, *C. africanus* KRAM F-57386 and KRAM F-57385, *Me. miltonrushii* MCA3882, *Tilletiaria anomala* UBC591, and *Tilletiopsis washingtonensis* MCA4186 (Tables 4, S2)*.* Second, screening steps are required to remove noise and artefacts from discovered variants. Good practices include masking variant sites observed from a single clone (Ganley and Kobayashi 2007; Lindner and Banik 2011), setting a minimum frequency cutoff for calling valid variant sites (Colabella et al. 2021), and integrating data from multiple methods. Comparison across the three detection methods in the *Ceraceosorus* ITS sequences from our study are beneficial for classifying detected variants whether they are real, artefactual, or ambiguous signals (Tables 4, S2). Finally, detailed methods for variant detection must be clearly stated, as they affect the validity of reported variant data. We recommend reporting all aspects of intragenomic variation (as discussed above) whenever possible. Having a standard convention for reporting sequence heterogeneity will be valuable for comparative analyses across studies to expand the breadth of knowledge of fungal rDNA evolution.

## CONCLUSION

In summary, our study describes the new species of *Ceraceosorus americanus*, representing the first report of *Ceraceosorus* from the Western Hemisphere. The recalcitrant growth of *Ceraceosorus* in culture and the lack of ITS barcode for this genus, due to ITS sequence heterogeneity, raise concerns that there might be other fungi in nature that are completely undetected by conventional methods. Through various sequencing and detection methods, we find extensive intragenomic variation of rDNA regions in all *Ceraceosorus* species/samples, but not in other *Ustilaginomycotina* lineages. Therefore, the genus *Ceraceosorus* can serve as a promising model to study relaxed concerted evolution in the rDNA region through comparative genomics and experimental genetics. We also notice result inconsistency across different variant detection methods. Pros/cons and recommendations for each detection method are detailed and discussed. We anticipate that our results will have implications for future research in taxonomy, systematics, metagenomics, evolutionary biology, genomics, and any other disciplines that uses rDNA as a tool for study.

## Supplementary Information

Supplementary material 1. Supplementary Tables

Supplementary material 2. Supplementary Figures

## Data Availability

Reference rDNA sequences and ITS clone sequences generated from this study are available in the NCBI GenBank database under the accessions provided in Table 1. WGS sequencing data used in this study were retrieved from the DOE-Joint Genome Institute (JGI) Fungal Genomics Program and will be available upon request. Other targeted amplicon high-throughput sequencing data, including bioinformatic scripts used in the analysis are available in the DRYAD repository (https://doi.org/10.5061/dryad.qbzkh18r3).

## Abbreviations

rDNA: Ribosomal DNA
ITS: Internal transcribed spacer
WGS: Whole-genome shotgun
PDA: Potato dextrose agar
CMA: Corn meal agar
YMA: Yeast malt agar
YPGA: Yeast peptone glucose agar
YMB: Yeast malt broth
