# Supplementary material for: Elucidation of intragenomic variation of ribosomal DNA sequences in the enigmatic fungal genus Ceraceosorus, including a newly described species Ceraceosorus americanus
Source: IMA Fungus. 2024 Dec 30;15:42. doi: 10.1186/s43008-024-00172-7 (PMC11687029; doi:10.1186/s43008-024-00172-7)
Supplement: Supplementary file 2 — Supplementary material 2. Supplementary Figures [file 43008_2024_172_MOESM2_ESM.docx]

**Supplementary Figures**

The enigmatic fungal genus *Ceraceosorus* reveals theoretical framework in studying intragenomic variation of ribosomal DNA sequences

Teeratas Kijpornyongpan^1^, Mary Claire Noble^1^, Marcin Piątek^2^, Matthias Lutz^3^ and M. Catherine Aime^1^*

^1^Department of Botany and Plant Pathology, Purdue University, West Lafayette IN, USA

^2^W. Szafer Institute of Botany, Polish Academy of Sciences, Kraków, Poland

^3^Department of Plant Evolutionary Ecology, Institute of Evolution and Ecology, University of Tübingen, Germany

*Corresponding author: maime@purdue.edu

**
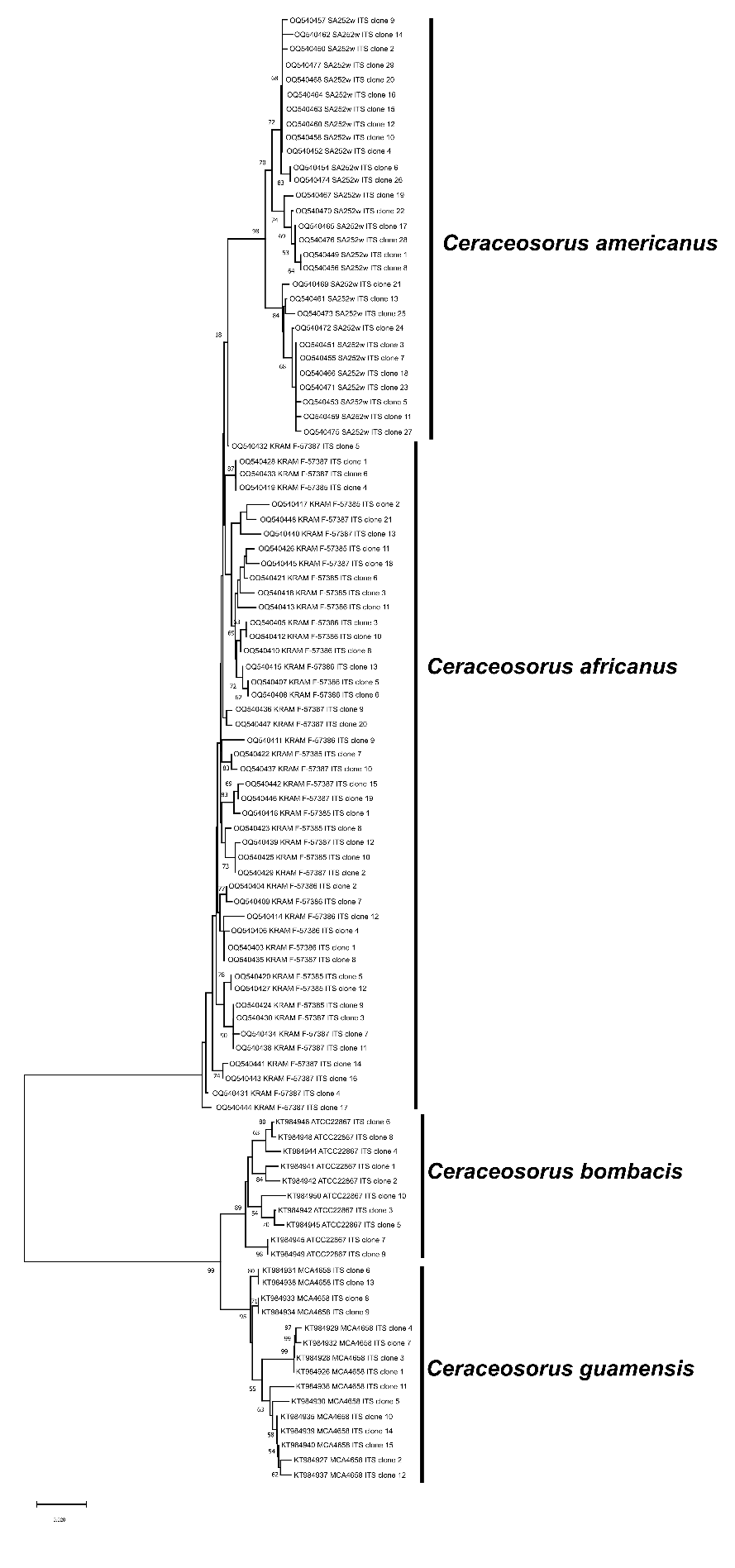
**

**Figure S1** Neighbor-joining tree of original ITS clone sequences from *Ceraceosorus.* The ITS amplicons from each *Ceraceosorus* sample were cloned using T-vector and sequenced through the Sanger platform. Resulting DNA sequences were used for MUSCLE alignment and phylogenetic tree reconstruction through the neighbor-joining method. Each tip indicates GenBank sequence accession, strain/ specimen number, and sampled clone number. Each node indicates a support value (only the value > 50% is shown) from 1000-replicate bootstrapping. Line labels show boundaries of clone sequences sampled from the same species. Bar: 0.02 substitutions per site.


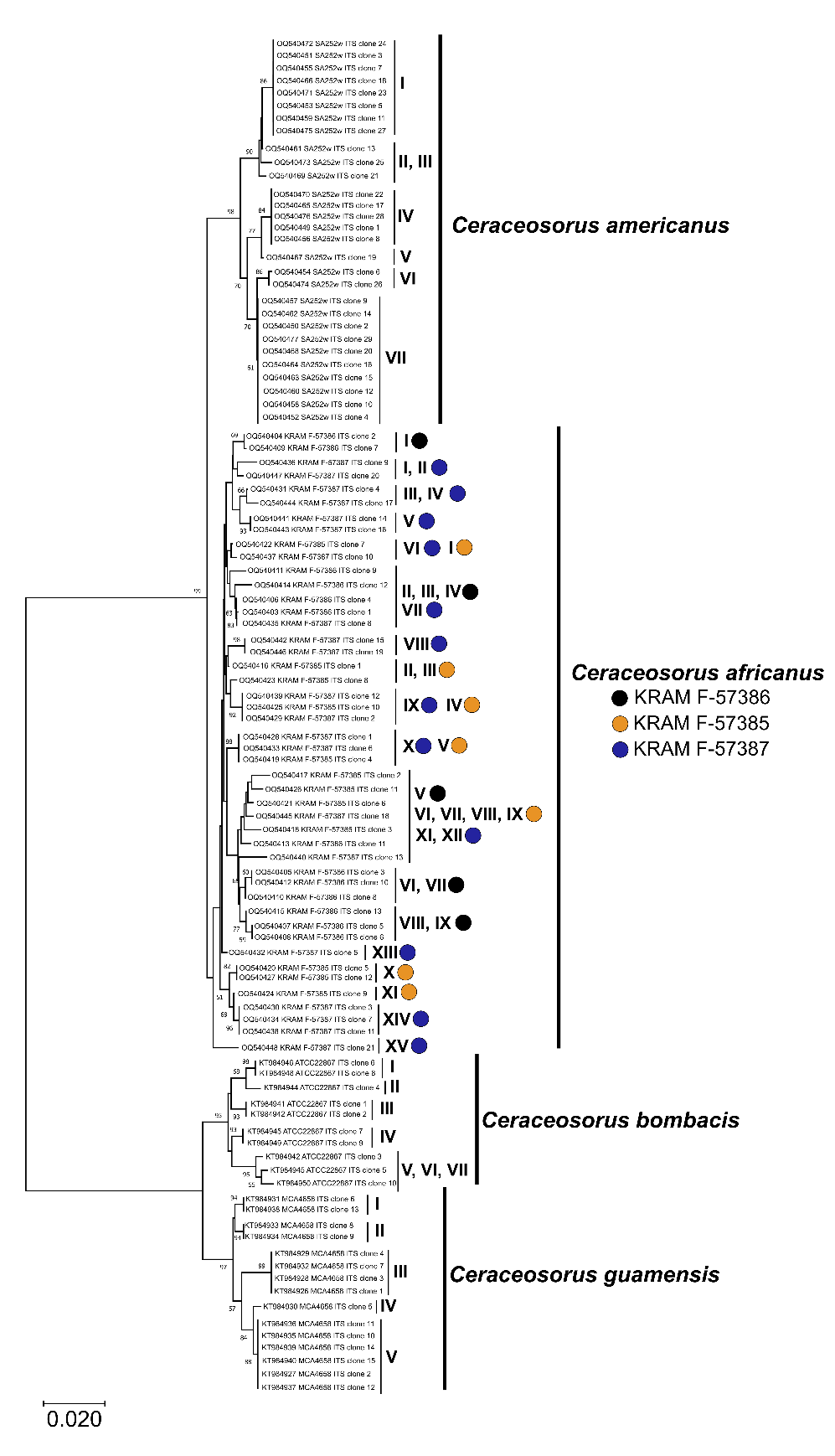
**Figure S2** Neighbor-joining tree of *Ceraceosorus* ITS clone sequences after PCR-sequencing error correction*.* The original ITS clone sequences were aligned separately for each sample. Then, we cross-checked the validity nucleotide variant sites in each alignment by comparing with variant discovery from targeted-amplicon sequencing and/or WGS sequencing. The variant sites present only in one clone for each sample but not in other variant discovery methods were considered as errors from PCR-sequencing. The ITS clone sequences from all *Ceraceosorus* samples were corrected accordingly before alignment and phylogenetic tree reconstruction. Each tip indicates GenBank sequence accession, strain/ specimen number, and sampled clone number. Each node indicates a support value (only the value > 50% is shown) from 1000-replicate bootstrapping. Line labels show boundaries of clone sequences sampled from the same species. Labels with roman numbers indicate haplotype numbers counted for each species. Colored circles indicate multiple specimens from *C. africanus*. Bar: 0.02 substitutions per site.


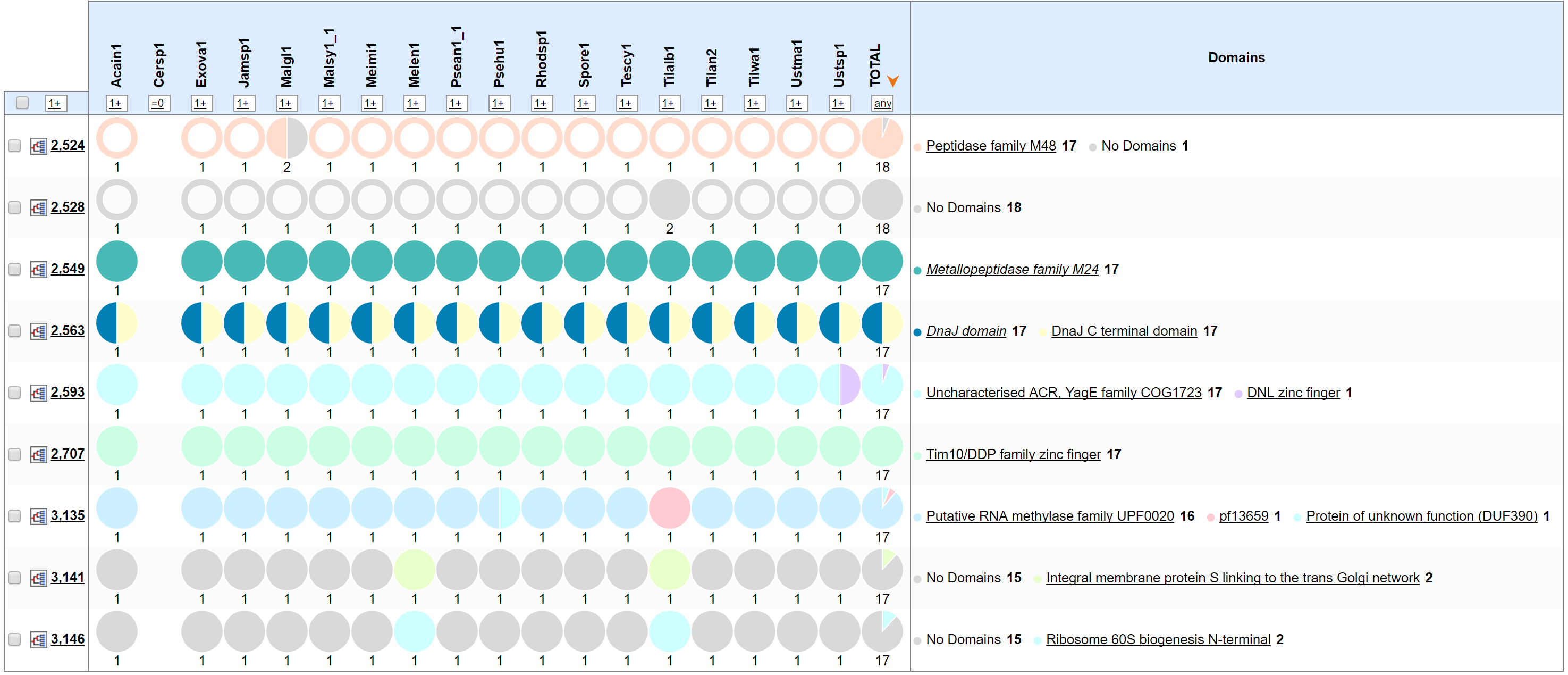


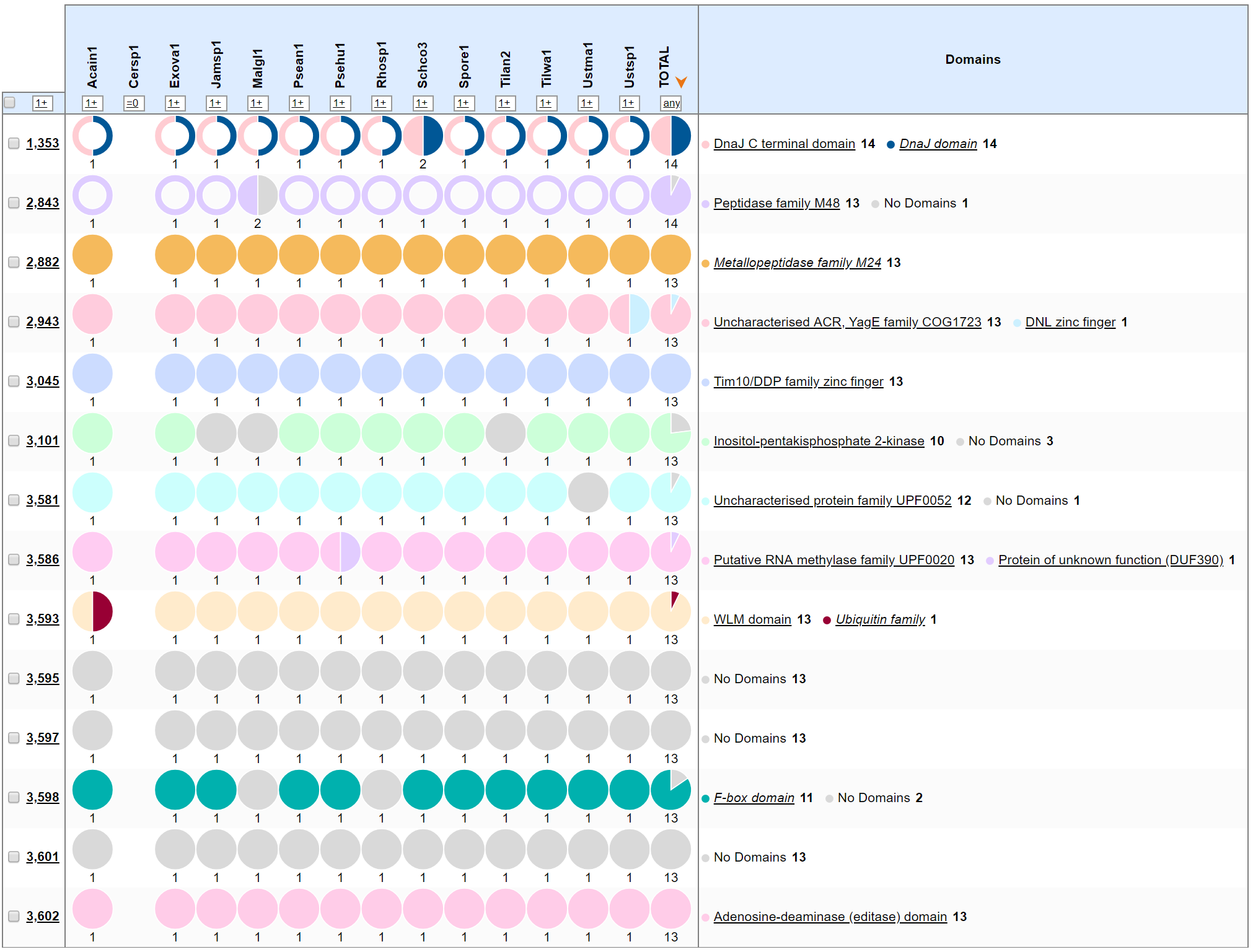


**Figure S3** Gene presence/absence matrix of orthologous genes among several Ustilaginomycotina genomes. Gene orthology data were retrieved from the JGI MycoCosm fungal portal under the ‘MCL cluster’ tab in the subphylum Ustilaginomycotina (retrieved in August 2021). Only genes that are absent in *Ceraceosorus guamensis* but present in other Ustilaginomycotina species are present in this figure. Abbreviations: Acain1, *Laurobasidium hachijoense* (syn. *Acaromyces ingoldii*); Cersp1, *Ceraceosorus guamensis*; Exova1, *Exobasidium vaccinii*; Jamsp1, *Jaminaea rosea*; Malgl1, *Malassezia globosa*; Psean1, *Moesziomyces antarcticus* (syn. *Pseudozyma antarctica*); Psehu1, *Pseudozyma hubeiensis*; Rhosp1, *Rhodotorula* sp.; Schco3, *Schizophyllum commune*; Spore1, *Sporisorium reilianum*; Tilan2, *Tilletiaria anomala*; Tilwa1, *Tilletiopsis washingtonensis*; Ustma1, *Mycosarcoma maydis* (syn. *Ustilago maydis*); Ustsp1, *Violaceomyces palustris*.
